# Mutations in the N-glycan branching enzyme MGAT1 promote invasiveness and EGF-stimulated proliferation and adhesion in neuroblastoma cells

**DOI:** 10.1371/journal.pone.0358077

**Published:** 2026-09-10

**Authors:** M. Kristen Hall, Lyla J. Walker, Abel R. Messer, Ruth A. Schwalbe

**Affiliations:** Department of Biochemistry and Molecular Biology, Brody School of Medicine, East Carolina University, Greenville, North Carolina, United States of America; University of Mississippi, UNITED STATES OF AMERICA

## Abstract

Attachment and modification of N-glycans to proteins have been linked to the promotion of cancer, and specifically, more recent investigations have supported the involvement of less processed N-glycans. Herein, we investigated how reduced activity of N-acetylglucosaminyltransferase-I (GnT-I, *MGAT1*) influences aberrant cellular properties and epidermal growth factor receptor (EGFR) signaling in human NB cells. CRISPR/Cas9 technology was used to introduce mutations into *MGAT1* in the parental BE(2)-M17 cell line, generating the *MGAT1* mutant cell lines, Trunc and Δ45. Lectin binding studies showed increased oligomannose type N-glycans accompanied by decreased hybrid and complex types of N-glycans in both *MGAT1* mutant cell lines relative to BE(2)-M17, and the level of oligomannose type N-glycans were reversed in the mutant cells by ectopic expression of GnT-I. Reduced GnT-I activity increased the percentage of S-type cells and decreased the percentage of N-type cells. Anchorage-independent cell growth was reduced in both *MGAT1* mutant cell lines, and cell proliferation was slower at sub-confluency. Unexpectedly, cell proliferation rates were increased in confluent and over-confluent cell cultures, as they were in three-dimensional cell spheroids, in the *MGAT1* mutant lines compared to the parental cell line. Trunc and Δ45 cells were less sensitive to mechanical dissociation and retained larger intact cell clusters, supporting that increased oligomannose N-glycans strengthen cell-cell adhesion. *MGAT1* mutant cell lines were also more invasive than BE(2)-M17. Following EGF stimulation, Trunc and Δ45 cells had increased levels of P-EGFR (Y1068), along with enhanced EGF-stimulated cell proliferation and cell-cell adhesion, whereas the parental cell line was relatively unresponsive. Taken together, these results show that decreased GnT-I activity imparts NB cells to perpetually proliferate at high cell densities. Furthermore, increased oligomannose N-glycans strengthened cell-cell contacts, promoted cell invasiveness, and enhanced EGF-stimulated proliferation, supporting a novel role of oligomannose N-glycans in NB progression.

## Introduction

Neuroblastoma (NB) is a heterogeneous pediatric malignant tumor and is attributed to around 15% of childhood cancer deaths [1]. The evidence of the heterogeneity of NB is demonstrated in the prognosis of patients, as low and intermediate risk NB patients have higher survival rates, while high-risk NB patients are afforded limited treatment options and often relapse [2]. Treatment modalities vary dependent upon the stage of the disease, while the more aggressive treatments for high-risk NB yield long-term side effects detrimental to development and quality of life for children [3]. Unmistakably additional diagnostics and therapeutics are needed for the children suffering from NB.

N-glycosylation is worthy of examination as glycan modifications on the surface of cells have been associated with cancer progression [4]. N-glycosylation is the most complex co-/post-translational protein modification in which glycans, and monosaccharides are systematically added to proteins [5]. The three primary types of N-glycans are oligomannose, hybrid, and complex, and they each have various monosaccharides/glycans linked to a common pentasaccharide core. The least processed oligomannose N-glycans are converted to hybrid type via N-acetylglucosaminyltransferase-I (GnT-I; *MGAT1*), and subsequently hybrid type to complex type via N-acetylglucosaminyltransferase-II (GnT-II; *MGAT2*). Additonal branch points are initiated by other GnTs and enhance the structural diversity of N-glycosylated proteins [5]. The ability to unravel this structural complexity of N-glycans and establish associations of specific N-glycan types to their functions in cancer progression is worthy of further research.

Mutations or overexpression of the Epidermal Growth Factor Receptor (EGFR), a transmembrane tyrosine kinase receptor, has been observed in a wide range of malignant tumors [6,7], including NB [8–10]. Moreover, progression and metastasis of NB significantly increased from overexpression of EGFR due to a decrease in cell apoptosis and stimulation of growth pathways [11]. EGFR is heavily N-glycosylated, including numerous highly processed N-glycans (e.g., complex type N-glycans) and utilization of the N-glycosylation sites are critical for EGFR structure/function [12]. Chemical inhibition to halt utilization of N-glycosylation sites significantly reduced EGFR signaling [12–15] while chemical inhibition used to prevent conversion of oligomannose to hybrid was not a necessity [14,16,17]. In contrast, a later study showed that the lowest processed type of N-glycan, oligomannose, did not support cell surface localization of ectopically expressed EGFR in a Chinese hamster ovarian (CHO) cell line which lacked GnT-I activity [18]. Our recent study agreed with the chemical inhibition study that EGFR associated with oligomannose type N-glycans is at the cell surface using 2D or 3D *MGAT1* mutant cell cultures [19]. While it is known that the N-glycans of EGFR influence its structure/function and thus downstream signaling [19–22], and EGFR is a therapeutic target of various cancers [10,12], gaining a better understanding of how various types of N-glycans attached to EGFR impact cancerous cell properties would be instrumental to the field.

N-glycosylation sites of EGFR are primarily occupied by complex N-glycans and only a couple oligomannose N-glycans [12]. Since oligomannose N-glycans are highly expressed in a variety of cancer cell lines [23], including NB [24–26], and have been associated with the progression of various cancers such as breast, ovarian, liver, and prostate cancer [27–30], it is of interest to establish the role of increased oligomannose N-glycans in NB cell lines on cellular properties, including EGFR signaling. Recently, we have shown that human and rat NB cells with enriched oligomannose N-glycans were more invasive, suggesting oligomannose N-glycans are indicators of aggressive NB [24,31]. Furthermore, we demonstrated that NB cells with elevated levels of oligomannose N-glycans were sensitized to EGF stimulation, as EGFR autophosphorylation and cell proliferation were heightened [19].

The objective herein is to further substantiate the impact of oligomannose type N-glycans on promoting invasiveness and growth in human NB cell lines using CRISPR/Cas9 technology [32] to generate two independent clonal cell lines with genetic edits to the *MGAT1* gene (lessened GnT-I activity), referred to as ∆45 and Trunc. Both clonal cell lines expressed heightened oligomannose type N-glycans relative to the parental BE(2)-M17 cell line, as demonstrated via lectin binding, while the parental line revealed heightened complex type. Cell morphological studies revealed variations in the population of cell types among the cell lines due to reduced GnT-I levels. Moreover, cell proliferation and anchorage independent growth were diminished in the mutant lines relative to the parental when examined at low confluency, while proliferation rates were increased at higher confluency, corroborating an association between cell type and cell growth/proliferation. Notably, rescue of the mutant cell lines via transient transfection with *Mgat1* verified on-target effects since GNL binding was decreased in *MGAT1* mutant cell lines overexpressing GnT-I, and proliferation rates of the mutant lines increased closer to that of the parental cell line. Likewise, cells with higher levels of oligomannose type N-glycans revealed greater cell invasiveness and cell-cell adhesion. The endogenous expression of EGFR was confirmed via western blotting; further, cell lines with high oligomannose type N-glycans showed enhanced levels of EGFR autophosphorylation relative to EGFR with complex N-glycans. Activation of EGFR signaling pathway with EGF resulted in a significant increase in cell proliferation and cell-cell adhesion in the mutant cells while the parental line was non-stimulated. Thus, the current study substantiates prior reports [24,25,33], ascertaining that oligomannose type N-glycans promotes NB development and progression by establishing the role of oligomannose on perpetuating cell proliferation at high cell densities, enhanced cell invasiveness, and heightened EGFR activation.

## Materials and methods

### Cell lines and cell culture

The human BE(2)C-M17 cell line, a neuroblastoma cell line, was purchased from American Type Culture Collection (Manassas, VA, USA). CRISPR/Cas9 technology was used to edit the α-1,3-mannosylglycoprotein-2-β-N-acetylglucosaminyltransferase (*MGAT1*) (accession:CR45681) and clones were selected in a similar manner as we previously described for the BE(2)-C cell line [19]. Genomic fragment sequencing verified that one expanded NB cell colony had 45 nucleotide deletion of residues located at about 2/3 of the length from the C-terminus of the protein and is referred to as the ∆45 cell line. DNA sequencing of the other expanded cell colony, referred to as the Trunc cell line, had a cell clone with 16 nucleotide deletion and another with an indel of 5 nucleotides. The indel had a nucleotide deletion and 4 nucleotide insertion. In both cases, the truncated protein (Trunc) synthesized lacked the last 75% of the amino acid residues. DNA sequencing was verified in nine bacterial cell colonies for each cell line. Both created cell lines were used to eliminate off-target effects, and to support on-target effects. Cells were sustained in complete DMEM (10% FBS, 50 U/mL penicillin, 50 μg/mL streptomycin) at 5% CO_2_ at 37 °C. Overexpression and rescue of *MGAT1* was attained using pCDNA3.1 vector containing the mouse *MGAT1* cDNA via Lipofectamine^®^ 3000 (Thermo Fisher Scientific, Rochester, MA, USA) protocol [24]. The *Mgat1* CDS was a kind gift from Dr. Pamela Stanley, College of Albert Einstein [34], and utilized for cloning into the PCDNA3.1 vector [24].

### 3D cell spheroid formation

The SPHERICALPLATE 5D® (Kugelmeiers, Erlenbach, Sweden) was used to create 3D cell spheroids as previously described [24]. In summary, the plate containing 0.5 mL of DMEM media per well was initially centrifuged (3 min/500 × *g*) to eliminate air bubbles. Next, cells were plated (7.5 × 10^4^ per well) and spheroids allowed to generate one, two, and four days in complete DMEM (50 U/mL penicillin, 50 μg/mL streptomycin, 10% FBS) at 37 °C, 5% CO_2._ Images were taken at days 1, 2, and 4 days using an Olympus CKX41 microscope 20X objective. Image J software, version 1.54d was used to quantify spheroid area.

### Whole cell lysates

Whole cell lysates were harvested in RIPA buffer (PBS, 1% Triton X-100, 0.5% sodium deoxycholate, 0.1% SDS, protease inhibitor cocktail set III) (EMD Biosciences, San Diego, CA, USA) as previously described [24]. In brief, cells were cultured to confluency on 35 mm dishes followed by harvesting using a cell scraper and resuspend in RIPA buffer. Cells were sheared using a 20 G needle, incubated on ice for 30 min, then centrifuged (15,000 × *g* / 20 min / 4 °C; Eppendorf F-45–30-11 rotor (Eppendorf, Westbury, NY, USA) and supernatant collected. Samples were kept at −80 °C until needed. Of note, for the overexpression of GnT-I, transiently transfected lysates were harvested at 24 hrs post transfection. Additionally, for the EGF treated lysates, confluent cells were sera starved for 22–24 hrs, followed by EGF treatment for two minutes prior to harvesting in RIPA buffer containing 2 × Protease/Phosphatase inhibitor (Thermofisher Scientific, Waltham, MA, USA).

### EGF treatment

In all assays using EGF treatment, cells were initially serum-starved for 22–24 h prior to treatment. Subsequently, cells were treated with or without 20 ng/mL recombinant human EGF (ThermoFisher Scientific, Waltham, MA, USA) and utilized in western blotting, proliferation and dissociation assays as described herein.

### Western and lectin blotting

Proteins were separated on Any kD™ Mini-PROTEAN® TGX™ gels (Bio-Rad, Hercules, CA, USA) and transferred to a PVDF membrane (Millipore, Billerca MA, USA), as previously described [24]. Lectin blot membranes were blocked with 3% bovine serum albumin (BSA), then incubated overnight in biotin-conjugated *Phaseolus vulgaris* erythroagglutinin (E-PHA), *Phaseolus vulgaris* leucoagglutinin (L-PHA), or *Galanthus nivalis* Lectin (GNL) lectins (Vector Laboratories, Burlingame, CA, USA). Western blot membranes were blocked with 5% PhosphBLOCKER™ (Cell Biolabs, Inc., San Diego, CA, USA), then incubated overnight in primary antibodies β-actin (Abcam, Cambridge, MA, USA), Vimentin, EGFR, or P-EGFR-Y1068 (Cell Signalling Technology, Danvers, MA, US). Membranes were washed three times (lectins, 1X PBS + 1% Tween20) or (westerns, 1X TBS + 1% Tween 20), followed by incubation with streptavidin (lectins) (Vector Laboratories, Burlingame, CA, USA) or specific alkaline phosphate conjugated secondary antibody (westerns). Blots were developed with 1-Step™ NBT/BCIP (Thermo Scientific, Rockford, IL, USA). Image J 1.54d software was utilized for analysis of western blots. Quantification of band intensity per lane was normalized to total protein loading via β-actin levels.

### Cell morphology

Cells were plated at low density onto CellBind Culture dishes (Corning, NY, USA) and incubated for forty-two hours and images captured using a CKX41 microscope (Olympus, Tokyo, Japan) equipped with a 40 × objective. Cells were characterized as I-type, S-type, or N-type and the percentage of each cell morphological type per dish was recorded.

### Anchorage independent growth

The soft agar assay was used to assess anchorage-independent growth, as described previously [24]. In summary, a 1:1 mixture of 1% noble agar and 2X DMEM was dispensed onto a 35 mm dish and allowed to solidify for 30 minutes at room temperature. Next, cells (2.5 × 10^3^ cell/mL in DMEM) were combined (1:1) with melted, cooled 0.6% noble agar, mixed, and pipetted into wells with previously solidified agar layer, and allowed to solidify. DMEM (0.5 mL) was added on top of agar layers to avoid drying of agar layers. Colonies were allowed to form for 13 days. Post 13-day incubation, images were captured using an Olympus CKX41 microscope (40×), and the colony area was measured using Image J software, version 1.54d.

### BrdU proliferation

5-bromo-2-deoxyuridine (BrdU) (Millipore, Billerica, MA) internalization was used to assay cell proliferation, following the manufacturer’s protocol, as described previously [24]. In short, dispersed cells were plated onto a 96-well dish cell culture treated dish at low confluency (2 × 10^4^), confluency (4 × 10^4^), or over-confluency (7.3 × 10^4^). For 3D proliferation assessment, formed spheroids (1, 2, or 4 days) were pipetted into individual wells of culture plates. In all cases, cells were incubated with BrdU reagent for 22 h under standard culturing conditions. For the EGF treated samples, −/+ EGF (20 ng/mL) was added at the time of BrdU addition. Following the BrdU treatment, cells were fixed for 30 min at room temperature, incubated with an anti-BrdU monoclonal antibody, then incubated with a secondary goat-α-mouse IgG peroxidase conjugate. Absorbance was measured at 450 nm using a Multiskan FC plate reader (Fisher Scientific, Atlanta, GA, USA). The resulting absorbances (Abs) were averaged, and the resulting values were calculated as follows: average Abs ^+BrdU^ – average Abs ^−BrdU^ = relative rate of proliferation. The EGF-stimulated cell proliferation is represented as the ratio of the relative rate of proliferation for EGF treated cells to the mean of the proliferation for EGF untreated cells.

### Cell dissociation

Dissociation was carried out as previously described [24]. Briefly, cells were grown to a confluent monolayer on Cell Bind Culture dishes (Corning, NY, USA), rinsed twice with DMEM, and then detached from the dish via a cell scraper. Next, cells were dissociated by pipetting up and down seven times using a 1 mL pipette tip. EGF treated cells were incubated with EGF (20 ng/ml) for 22 hours prior to dissociating. Cell cluster images were captured using an Olympus IX71 microscope equipped with a 20 × objective. Area of Cell clusters (>10 cells) was measured using Image J software, version 1.54d. The ratio of cluster area for EGF treated cells to the mean of cell cluster area of untreated cells was used to report EGF-stimulated cell adhesion.

### 3D spheroid invasion

Cell spheroids (1 day formed) were collected in DMEM from SPHERICALPLATE 5D® (Kugelmeiers, Erlenbach, Sweden). Spheroids were allowed to settle, harvested, and pipetted into pre-cooled Matrigel (Corning, Corning, NY, USA), and mixed gently as previously noted [24]. A 40 μL drop of the spheroid-matrigel mix was pipetted into a 24-well dish and incubated (37 °C) for 30 minutes to allow the solidification of the matrigel. Next, complete DMEM (1 mL) was added to the well, and images were acquired to represent pre-invasion spheroid size. Spheroids were allowed to invade for 22 and 46 hours. Images of spheroid invasion were acquired using an IX71 Olympus microscope (20 × objective). The total area of pre-invading spheroid and invading spheroid at timepoints indicated were measured with Image J software, version 1.54d, and calculated: invasion area = (invading spheroid area – mean of non-invading spheroid area) / mean of non-invading spheroid area).

### Data analysis

Images for figures were prepared using Adobe Photoshop. Graphics and statistics were obtained using Origin 2026. Data are reported as the mean ± standard error of the mean (SEM). Where shown, “*n*” denotes the number of observations as indicated. Unpaired Student’s *t*-test was used to compare two groups, while one-way ANOVAs with the post hoc Holm–Bonferroni means comparison test was used to compare more than two groups.

## Results

### Construction of N-glycosylation mutant NB cell lines with introduction of indels in MGAT1

**Fig 1:**
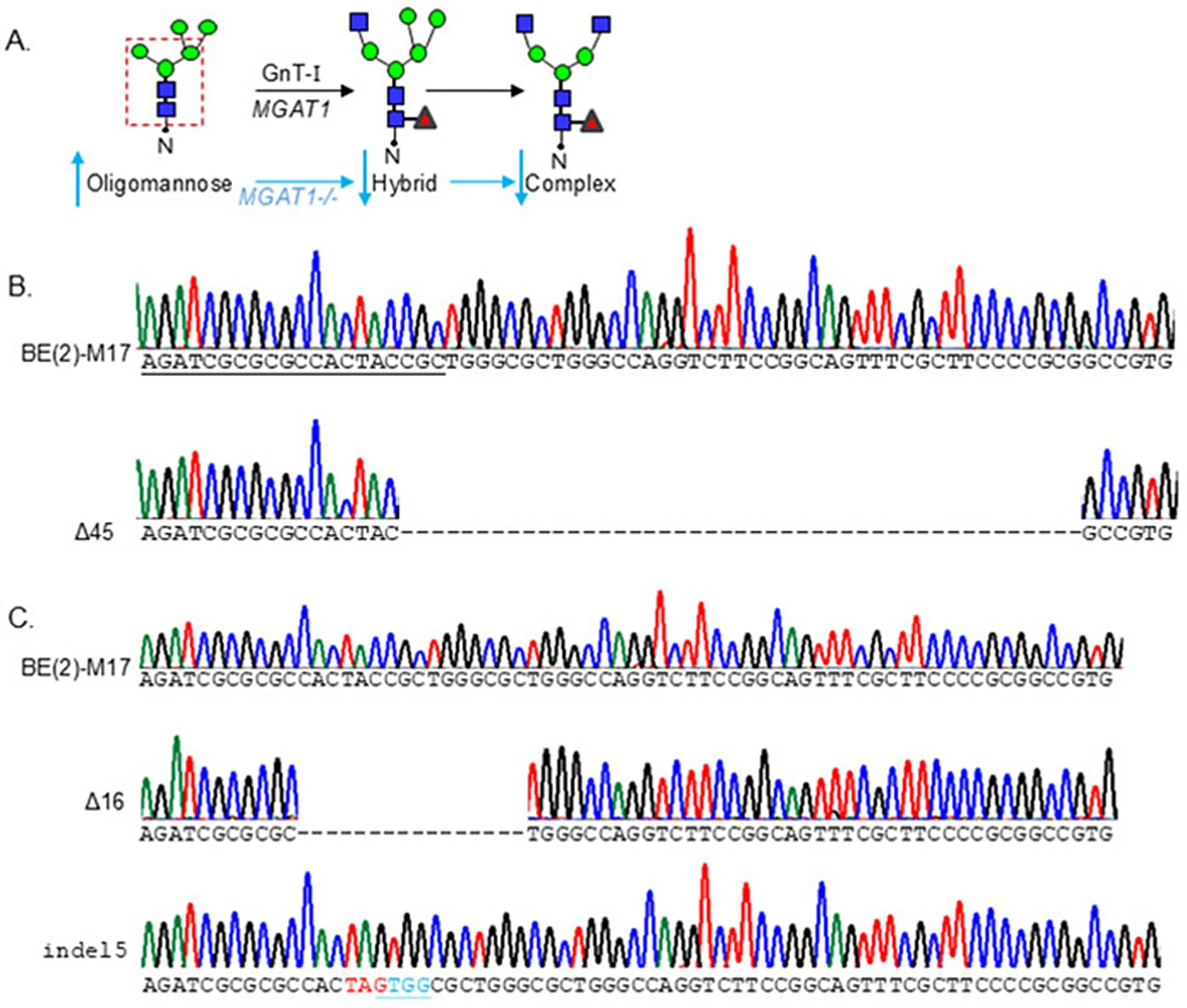
DNA chromatograms of a fragment of the *MGAT1* gene from BE(2)-M17 and engineered BE(2)-M17 cell lines. Three general types of N-glycans, including oligomannose, hybrid and complex, are attached via asparagine (N) residues of N-glycosylation sites (Asn-Xxx-Ser/Thr) (A). GnT-I is required to convert oligomannose type to hybrid and complex types of N-glycans. The red broken lined square denotes the conserved pentasaccharide. Blue arrows and font denote relative abundancies of N-glycan types when *MGAT1* is mutated (MGAT1−/−). Oligomannose N-glycans rise while both hybrid and complex N-glycans decline. Indels in the *MGAT1* gene of two different expanded cell colonies were identified. Comparison of the DNA sequence of BE(2)-M17 with a single cell clone lacking 45 nucleotides (∆45) (B), and another expanded cell colony with two cell clones, one missing 16 nucleotides (∆16), and another with a 5 nucleotide indel (indel5) (C). The blue underline shows where 1 nucleotide was deleted and 4 nucleotides inserted. The nucleotides in red font show the stop codon produced by the indel. The ∆45 had 15 amino acids missing, while both ∆16 and indel5 resulted in truncated protein.

The *MGAT1* gene codes for the GnT-I enzyme which converts oligomannose type to hybrid type followed by formation of complex type of N-glycans (Fig 1A). As such, slowed activity of GnT-I will result in increased and decreased levels of oligomannose and both hybrid and complex N-glycans, respectively. Employment of CRISPR/Cas9 technology generated two independent cell lines using a parental cell line called BE(2)-M17. DNA sequencing of *MGAT1* from one of the expanded cell colonies revealed 45 nucleotide deletion (Fig 1B) while the other cell colony had two cell clones, including 16 nucleotide deletion (∆16) and five nucleotide indel, indel5 (Fig 1C). The earlier cell line, referred to as ∆45, produced GnT-I lacking 15 amino acid residues while the later cell line generates two different forms of truncated GnT-I which is called Trunc.

**Fig 2:**
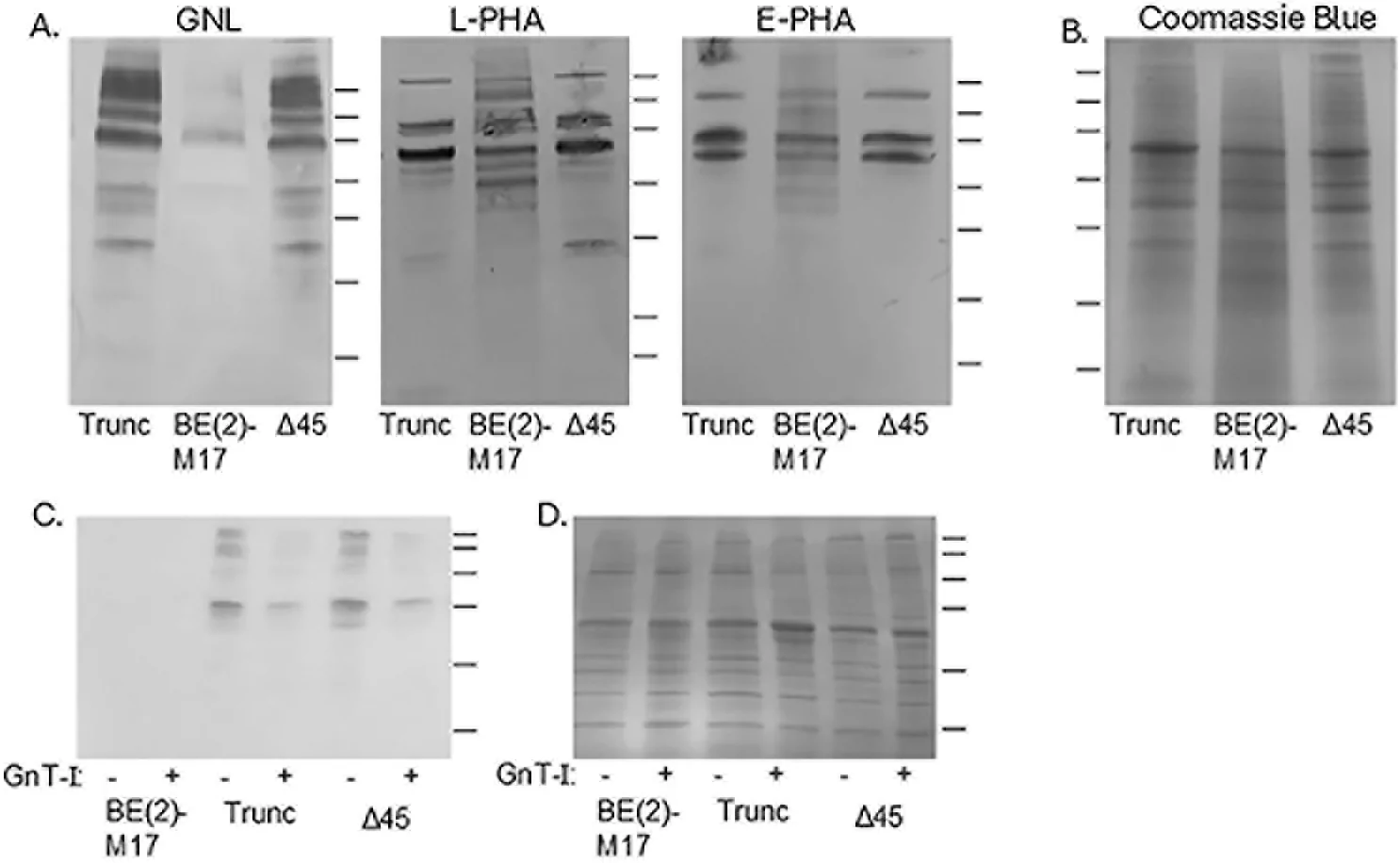
Lectin binding of glycoproteins in parental and glycosylation mutant cell lines. A) Whole cell lysates from Trunc, BE(2)-M17 and ∆45 cell lines. Separated N-glycosylated proteins from the various cell lines were probed with GNL, L-PHA, and E-PHA lectins. B) Coomassie blue-stained gel of total protein from the various cell lines. C) GNL probed blots of N-glycosylated proteins from whole cell lysates of BE(2)-M17, Trunc and ∆45 cells without (−) and with (+) expression of heterologous GnT-I, D) along with Coomassie blue-stained gel of the samples. Lines adjacent to membranes and gel denote molecular weights markers of 250, 150, 100, 75, 50, 37 and 25 kDa, except the lowest marker is not shown in panels C and D.

Three different lectins were used to probe the types of N-glycans attached to proteins in the parental and N-glycosylation mutant cell lines (Fig 2A). Proteins from whole cell lysates of BE(2)-M17, Trunc, and ∆45 cells were separated on a reducing SDS gel and probed with GNL, L-PHA, and E-PHA. GNL binds with high affinity to oligomannose N-glycans [35]. L-PHA has a high affinity for tri- and tetra-antennary complex N-glycans, while E-PHA has a high affinity for complex N-glycans with bisecting N-acetylglucosamine (GlcNAc) [35]. GNL binding was greatly increased for proteins isolated from Trunc and ∆45 cell lines compared to BE(2)-M17 (Fig 2A). It was also observed that L-PHA and E-PHA were bound to more glycosylated proteins from BE(2)-M17 relative to *MGAT1* mutant cell lines. The adjacent SDS gel stained with coomassie blue shows the amount of protein loaded for each cell line were quite similar (Fig 2B). When Trunc and ∆45 cell lines overexpressed *Mgat1* the level of GNL binding was reduced relative to those cells not overexpressing *Mgat1* (Fig 2C). GNL binding was quite similar in BE(2)-M17 cells with and without overexpression of *Mgat1*. Coomassie blue stained gel reveals similar levels of protein (Fig 2D). Thus, the lectin binding studies indicated that the edits in *MGAT1* which were identified by DNA sequencing had considerably decreased activity of GnT-I in ∆45 and Trunc cell lines relative to the parental cell line and that mutant cell lines could be rescued by ectopic expression of GnT-I.

### Decreased GnT-I activity altered NB cell morphology

**Fig 3:**
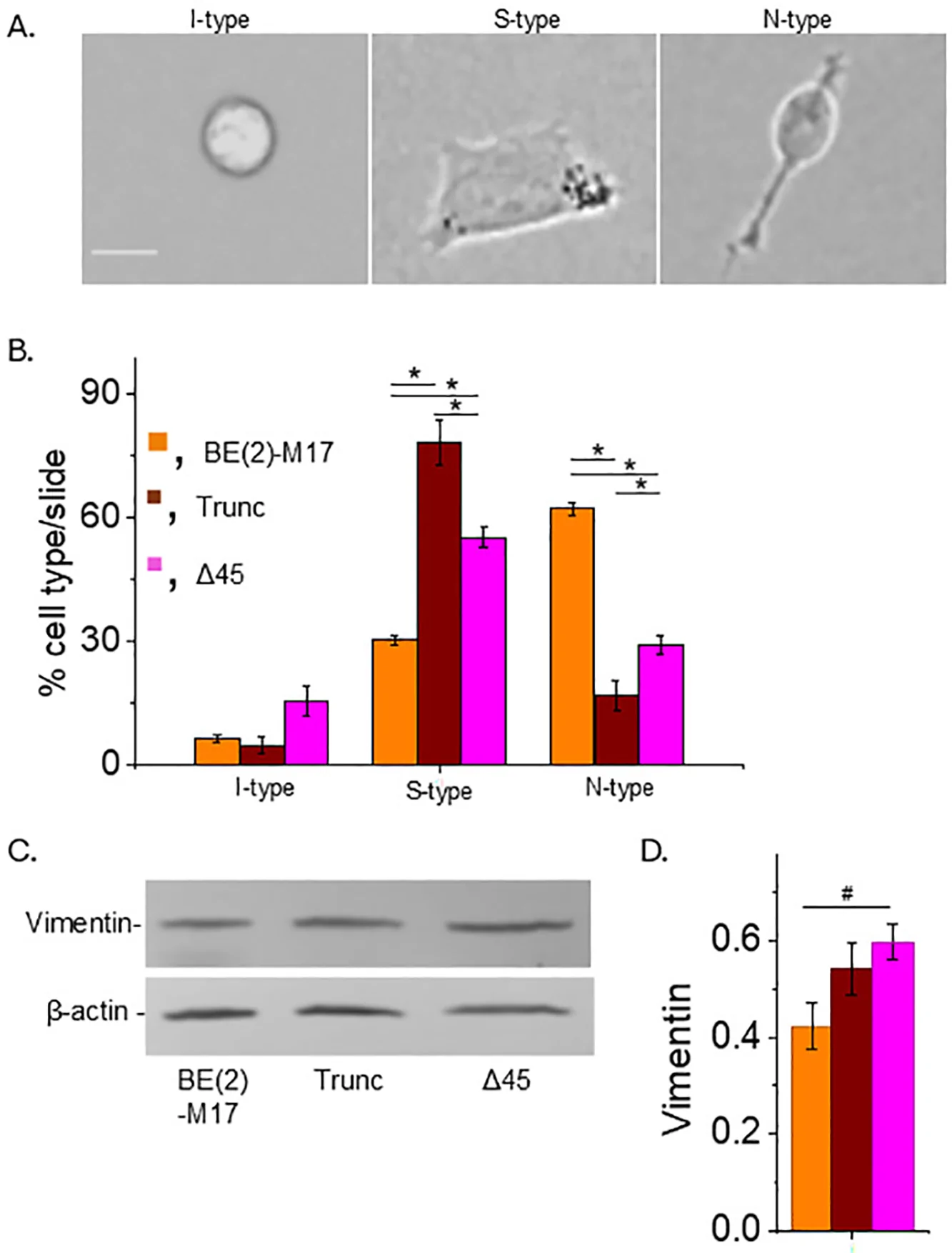
Variation of cell types between BE(2)-M17, Trunc, and ∆45 cell lines. A) I-type, S-type, and N-type cells were observed in all three cell lines. Scale bar is 10 µm. B) Percent of cell type per slide for BE(2)-M17 (*n* = 3), Trunc (*n* = 4), and ∆45 (*n* = 4) cell lines, *n* denotes number of slides. C) Western blot of vimentin, and D) quantification of vimentin immunoband with respect to β-actin from BE(2)-M17 (*n* = 9), Trunc (*n* = 6), and ∆45 (*n* = 6), where *n* denotes number of Western blots. Three biological samples were used. Numerical values are represented as mean ± SEM. One-way ANOVA with a post hoc Holm-Bonferroni was used to compare means, where *p < 0.05 and ^#^p < 0.1.

NB cell lines have been shown to be composed of different cell morphologies, including I-type, S-type, and N-type [36–38] (Fig 3A). Both *MGAT1*-/- cell lines, which had high oligomannose N-glycans, and low complex and hybrid N-glycans, showed increased levels of S-type, and decreased levels of N-type compared to BE(2)-C (Fig 3B). Moreover, the levels of S-type and N-type were higher and lower, respectively, in the Trunc cell line relative to the ∆45 cell line. Vimentin, a structural protein highly expressed in S-type cells [36–38], was observed in all three cell lines (Fig 3C and 3D). Vimentin levels were significantly different between BE(2)-M17 and ∆45 at p < 0.07, while it appeared the level in Trunc was more like ∆45 than BE(2)-M17. Neurofilament, a structural protein, often found in high levels in N-type cells [37,38], was not detected by Western blotting in the three cell lines (not shown). These results reveal that altering abundancies of the various types of N-glycans by lowered activity of GnT-I changes the expression of cell types in NB cells.

### Increased oligomannose N-glycans reduced non-adhered cell growth

**Fig 4:**
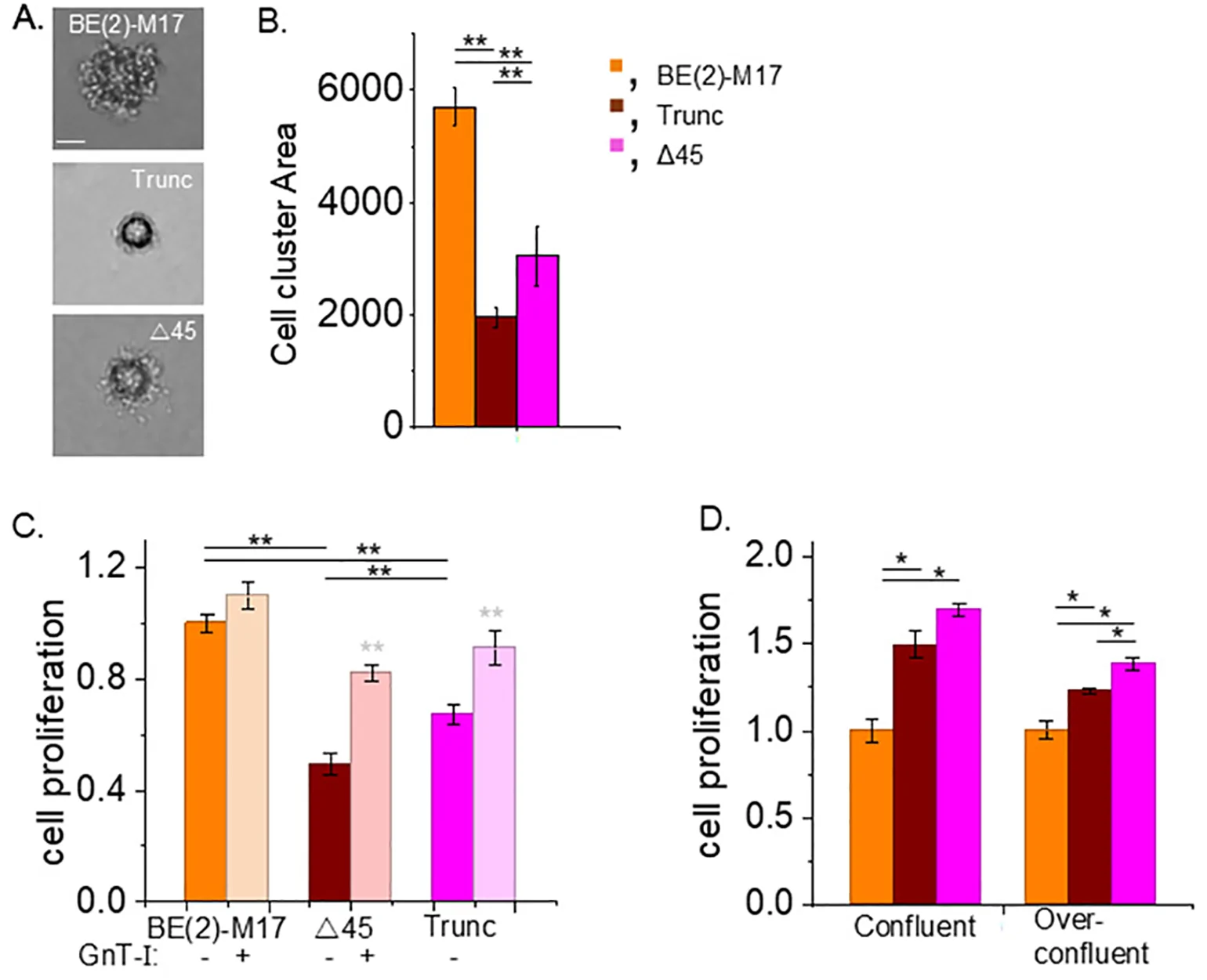
Deficient GnT-I cell lines had retarded non-adhered and adhered cell growth. A) Typical DIC images of cell colonies from anchorage independent growth of parental and N-glycosylated mutant cell lines. Scale bar is 5 µm. B) Cell growth was monitored by quantification of the area of cell colonies from BE(2)-M17 (*n* = 144), Trunc (*n* = 105), and ∆45 (*n* = 63) following 13 days of growth where *n* is the number of cell clusters. C) Sub-confluent cell monolayers of BE(2)-M17 (*n* = 13), Trunc (*n* = 9), and ∆45 (*n* = 9), along with BE(2)-M17 (*n* = 4), Trunc *n* = 4) (, and ∆45 (*n* = 4) ectopically expressing GnT-I were analyzed via BrdU cell proliferation assay. The fainter colors of BE(2)-M17 (■), ∆45 (■), and Trunc (■) represent cell lines overexpressing *Mgat1*. D) Adhered cells at confluency and over-confluency were monitored using the BrdU cell proliferation assay in (BE(2)-M17, Trunc, and ∆45 (*n* = 4). *n* denotes number of replicates. Data are presented as mean ± SEM. One-way ANOVA with a post hoc Holm-Bonferroni was used when comparing more than two samples, where *p < 0.05 and **p < 0.01. Student T-test was used to compare each cell line without and with GnT-I in panel C, where *p < 0.01.

The growth of BE(2)-M17, Trunc, and ∆45 cells without attachment and spreading onto a substratum was monitored by acquiring images after 13 days (Fig 4A). During this time course, both cell lines with deficient GnT-I displayed slower cell growth compared to BE(2)-M17 (Fig 4B). Thus, increased oligomannose type and decreased hybrid and complex types of N-glycans suppressed anchorage-independent cell growth.

### Raised oligomannose N-glycans impact adhered cell proliferation

Cell proliferation rates were measured via BrdU incorporation into the DNA during its replication process on cell culture plates at about 40% confluency. Cell proliferation was fastest in the BE(2)-M17 cell line and slowest in the Trunc cell line while ∆45 had an intermediate rate (Fig 4C). Moreover, when both mutant cell lines were transiently transfected with *Mgat1* cDNA, the cell proliferation rates were significantly increased, while that of BE(2)-M17 was virtually unchanged. These results showed that deficiencies in GnT-I significantly reduced cell proliferation. Moreover, transient expression of *Mgat1* in the glycosylation mutant cell lines raised proliferation rates to close to that of the parental cell line, and therefore strongly support that the decrease in proliferation rates of the mutant cell lines is due to deficient GnT-I activity.

Cell growth was measured at higher cell densities for BE(2)-M17, Trunc and ∆45 (Fig 4D). At confluency, proliferation rates of both mutant cell lines were significantly greater than those of the parental cell line. Above confluency, ∆45 had the fastest growth rate, followed by Trunc, while BE(2)-M17 had the slowest rate. Taken together, higher NB cell densities favor higher growth rates when GnT-I activity is lowered.

### Impact of deficient GnT-I on cell spheroid growth

**Fig 5:**
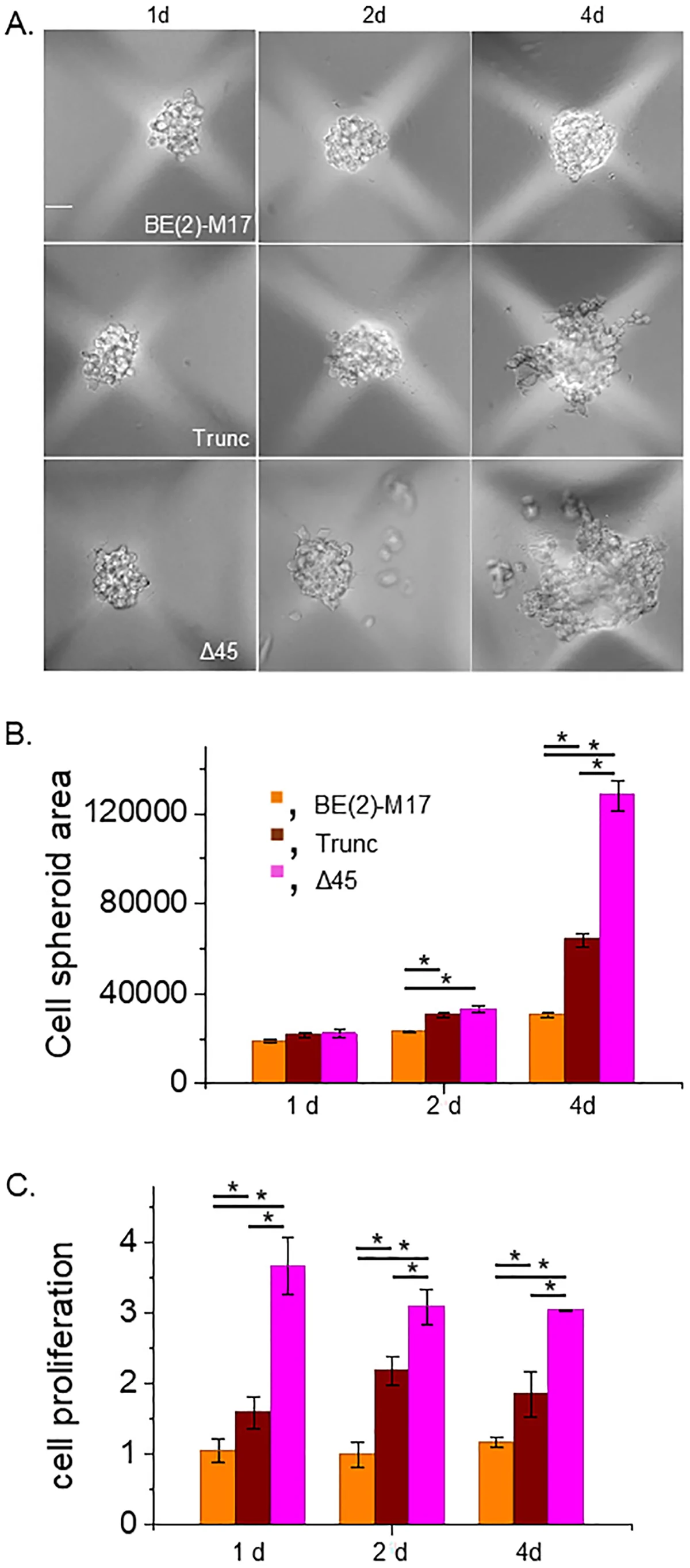
Cell spheroid formation and growth of parental and mutant cell lines. A) Representative micrographs of spheroids at 1, 2, and 4 days in culture. Scale bar is 25 µm. B) Quantification of spheroid area at 1,2, and 4 days from BE(2)-M17 (*n* = 78, 120, 62), Trunc (*n* = 79, 120, 61), and ∆45 (*n* = 81, 125, 68) cell lines. *n* denotes number of spheroids. C) Cell proliferation of 1, 2 and 4 days old spheroids (*n* = 3) for each sample, as indicated, where *n* is the number of replicates. Data is represented as mean ± SEM. One-way ANOVA with a post hoc Holm-Bonferroni was used to compare means, where *p < 0.05.

Micrographs of cell spheroids from BE(2)-M17, Trunc, and ∆45 cell lines were captured under 3D cell cultured conditions (Fig 5A). Cell spheroid size was quite similar at 1d for all three cell lines; however, at 2 d spheroid size was significantly smaller for BE(2)-M17 relative to ∆45 (Fig 5B). As 3D cell culturing continued (4 d), cell spheroid size was largest for ∆45 and smallest for BE(2)-M17 and intermediate for Trunc. To ascertain whether the increase in cell spheroid size was due to enhanced cell proliferation, the BrdU assay was performed using spheroids grown for 1, 2 and 4 d (Fig 5C). Cell proliferation was increased in both mutant lines from 1, 2, and 4 d spheroids relative to those of the parental cell line, furthermore, cell proliferation was greatest in the ∆45 cell line. Thus, *MGAT1* mutant cells begin to proliferate faster after 1 d spheroid formation/growth.

### Cell-cell adhesion is strengthened by increased oligomannose N-glycans

**Fig 6:**
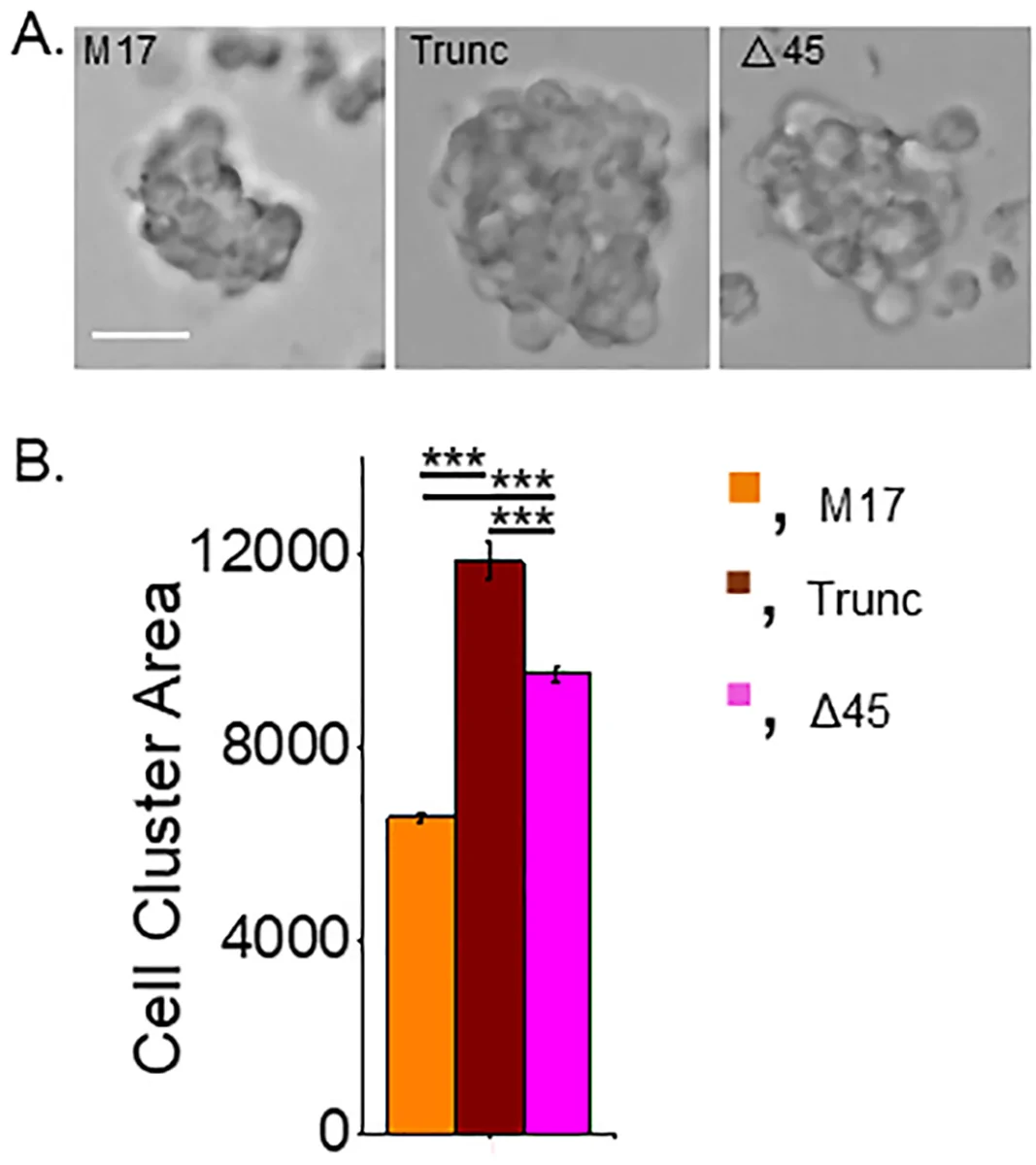
Lowered GnT-I activity enhances cell-cell adhesion. A) Illustrative microscopic images of intact cell clusters of BE(2)-M17, Trunc, and ∆45 after mechanical dissociation of a cell monolayer via pipetting. Scale bar is 25 µm. B) The area of cell clusters from BE(2)-M17 (*n* = 508), Trunc (*n* = 292), and ∆45 (*n* = 594). *n* denotes the number of cell clusters. Quantifications are signified as mean ± SEM. One-way ANOVA with a post hoc Holm-Bonferroni was used to compare means, where ***p < 0.0001.

Images of cell clusters were acquired for BE(2)-M17, Trunc and ∆45 cell lines following disruption of cell monolayers (Fig 6A). Overall, the cell clusters were larger when oligomannose N-glycans were more abundant. Quantification of the area of the cell clusters revealed a 1.8-fold increase for Trunc, and 1.5 -fold increase for ∆45 relative to BE(2)-M17 (Fig 6B). Hence, lowered GnT-I activity heightened cell-cell adhesion in NB cells.

### Promotion of cell invasiveness by decreased GnT-I activity

**Fig 7:**
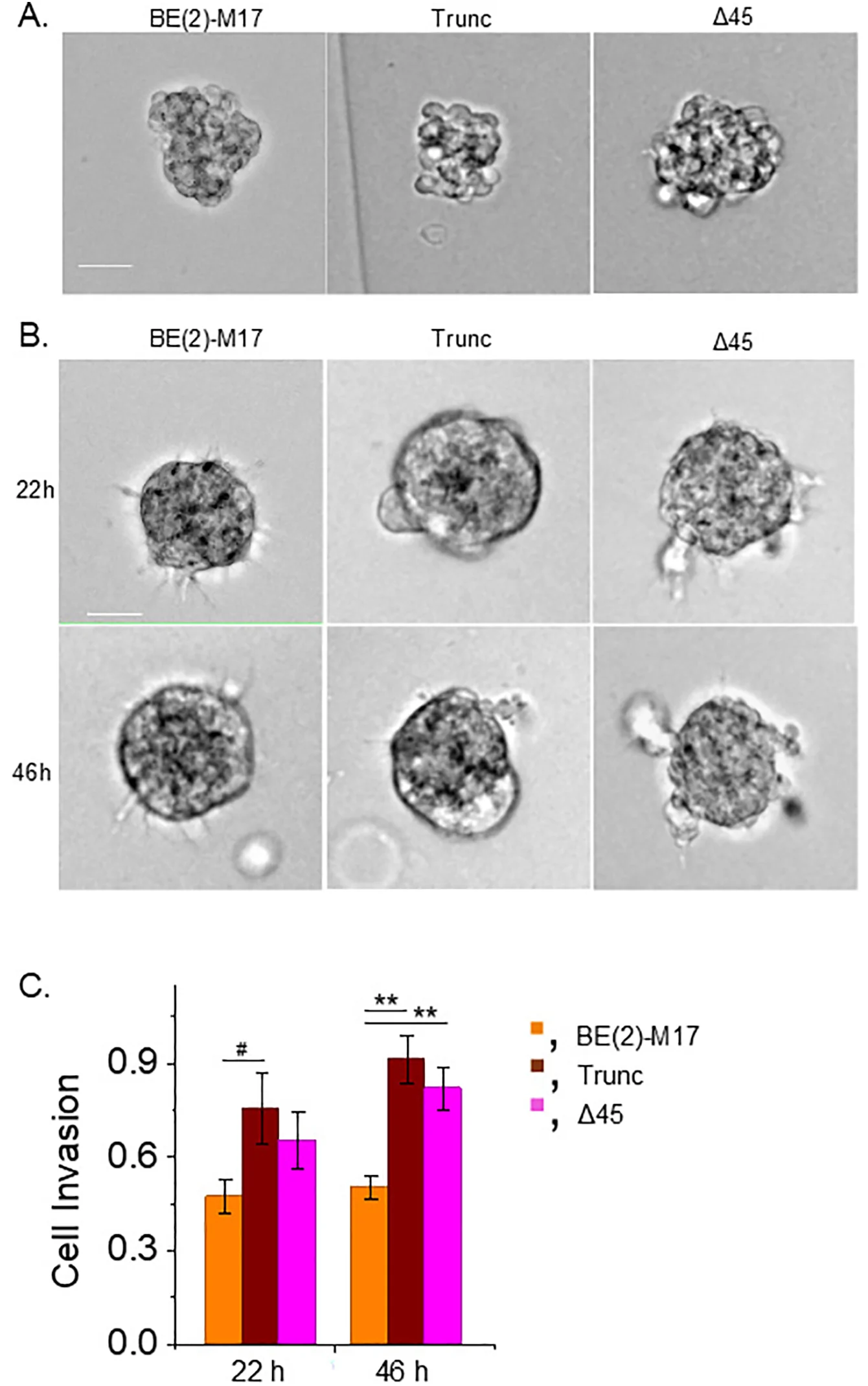
Loss of *MGAT1* promotes cell invasiveness. A) Typical images of pre-invasion spheroids. B) Representative micrographs of invading cell spheroids at 22 h and 46 h of the three cell lines, as indicated. Scale bars are 25 µm. C) The area of invading cell spheroid from BE(2)-M17 (*n* = 38, 44), Trunc (*n* = 37, 56), and ∆45 (*n* = 28, 54) at 22h and 46 h, respectively. *n* denotes the number invading cell spheroids. Quantifications are represented as mean ± SEM. One-way ANOVA with a post hoc Holm-Bonferroni was used to compare means, where **p < 0.01 and ^#^p < 0.1.

The three-dimensional tumor spheroid invasion assay was applied to evaluate cell invasiveness of BE(2)-M17, Trunc and ∆45 cell lines. Representative images of non-invading cell spheroids (Fig 7A) in matrigel, and those of invading spheroids at 22 h and 46 h (Fig 7B). The cell invasiveness of Trunc cell line was about 1.6-fold greater than BE(2)-M17 at 22 h (Fig 7C). As the culturing time of the invading spheroids in matrigel continued up to 46 h, both Trunc and ∆45 cell lines revealed increased invasiveness by 1.8-fold and 1.6-fold, respectively, relative to the parental cell line. Thus, these results support that oligomannose N-glycans promote cell invasiveness in NB cells.

### Heightened EGFR signaling by lowered expression of *MGAT1*

**Fig 8:**
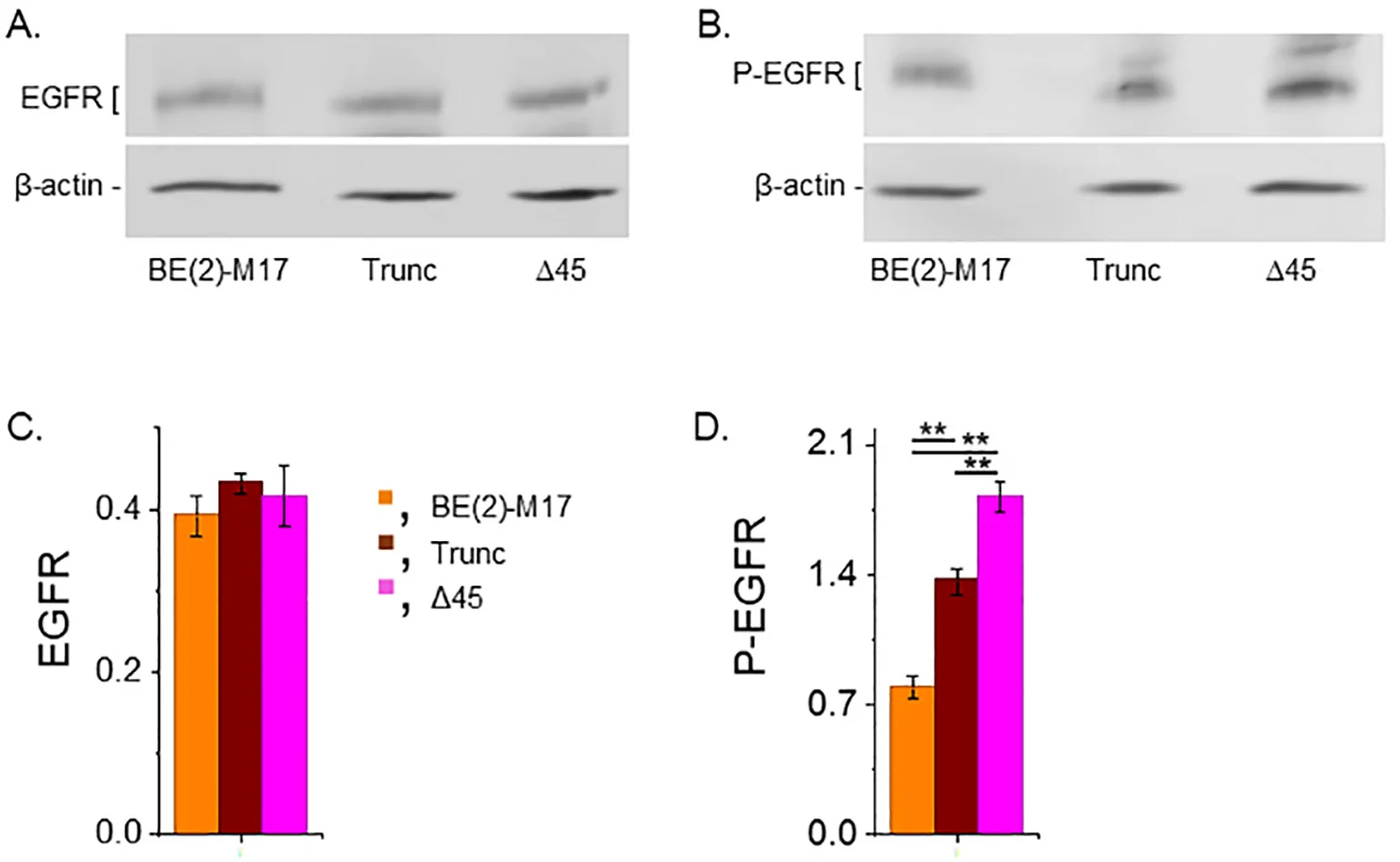
Reduced *MGAT1* expression alters phosphorylation of EGFR. A) Western blot of EGFR and B) phosphorylation of Y1068 in EGFR from whole cell lysates of BE(2)-M17, Trunc and ∆45. C) Quantification of EGFR and D) phosphorylated EGFR immunobands with respect to that of β-actin for BE(2)-M17 (*n* = 3), Trunc (*n* = 4), and ∆45 (*n* = 4), where *n* denotes number of immunobands. Cells were stimulated with 20 ng/mL EGF. Quantifications are represented as mean ± SEM. One-way ANOVA with a post hoc Holm-Bonferroni was used to compare means, where *p < 0.01. P-EGFR immunobands were unobserved in samples without EGF which were run in parallel to samples with EGF (S1 Fig).

Activation of EGFR signaling pathway by incubation of BE(2)-M17, Trunc, and ∆45 with EGF (20 ng/mL) was evaluated by Western blotting with EGFR (Fig 8A), along with phospho-EGFR (Y1068), referred to as P-EGFR) (Fig 8B). The immunoband intensities of EGFR were quite similar in the BE(2)-M17, Trunc, and ∆45 whole cell lysates while P-EGFR was observed to be less abundant in BE(2)-M17 than those of the *MGAT1* mutant samples. P-EGFR immunobands were not detected in samples from cells untreated with EGF (S1 Fig). The electrophoretic mobility of the EGFR immunobands of the *MGAT1* mutant samples were slightly faster than those of the BE(2)-M17, suggesting that the glycosylated forms of EGFR in the parental and N-glycosylated mutant cell lines were different, as we previously showed for parental and *MGAT1* mutant BE(2)-C cell lines [19]. This small mobility shift can be clearly viewed in S1 Fig. Quantification of the intensity of the EGFR immunobands with respect to β-actin showed levels of EGFR were similar between all three samples (Fig 8C), while intensity of the P-EGFR immunobands with respect to β-actin showed higher levels of EGFR autophosphorylation in both Trunc and ∆45 relative to BE(2)-M17 with the highest level of P-EGFR in ∆45 (Fig 8D). Hence, these results showed that EGFR in the presence of high oligomannose levels could more readily undergo autophosphorylation.

**Fig 9:**
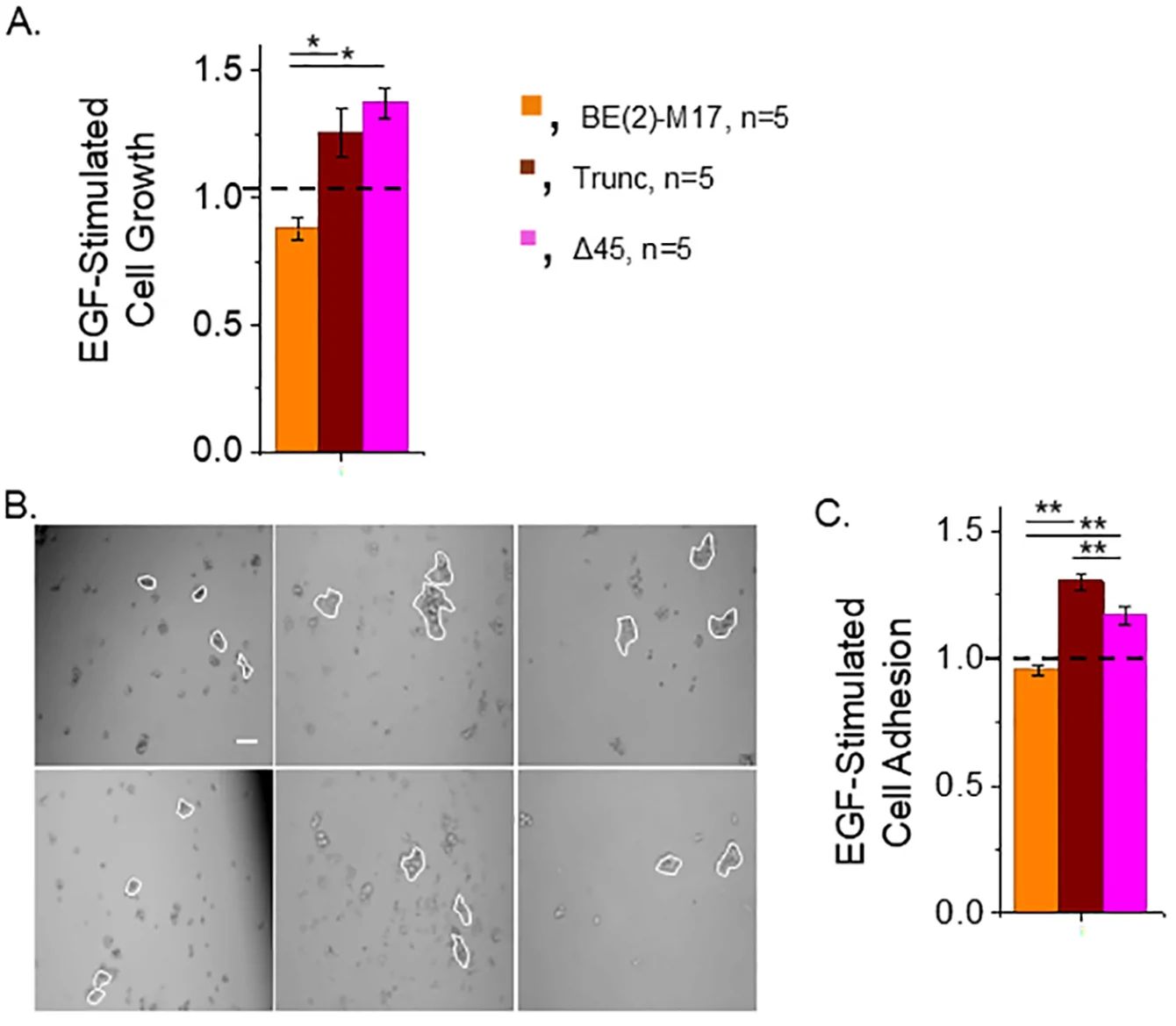
EGF treatment enhanced cell proliferation and cell adhesion in the *MGAT1*-/- cell lines. A) EGF-stimulated cell proliferation of BE(2)-M17 (*n* = 5), Trunc (*n* = 5), and ∆45 (*n* = 5) where *n* is the number of replicates. B) Micrographs of BE(2)-M17, Trunc, and ∆45 cell clusters with (top row) and without (bottom row) 20 ng/mL EGF stimulation, C) and quantification of EGF-stimulated cell-cell adhesion for BE(2)-M17 (*n* = 140), Trunc (*n* = 175), and ∆45 (*n* = 160) using 20 ng/mL, where *n* is the number of cell clusters. Scale bar is 25 µm. Data are expressed as mean ± SEM. One-way ANOVA with a post hoc Holm-Bonferroni was used to compare means from three samples and student t-test was used for two samples, where *p < 0.05, **p < 0.01.

Cellular responses due to EGFR signaling were evaluated in the parental and N-glycosylation mutant cell lines. BE(2)-M17 cells treated with and without EGF (20 ng/mL) had similar cell proliferation rates while the rates of both *MGAT1*-/- mutants, Trunc and ∆45, were significantly increased by EGF treatment (Fig 9A). Representative images of intact cell clusters post disruption of cell monolayer of BE(2)-M17, Trunc, and ∆45 without (bottom panels) and with (top panels) EGF (Fig 9B). EGF-stimulated cell-cell adhesion increased by at least 1.2 in the *MGAT*-/- cell lines while the parental cell line was unresponsive (Fig 9C). Cell clusters were larger when oligomannose N-glycans were more abundant. Taken together, these results show that higher levels of oligomannose N-glycans in NB cells heighten EGFR autophosphorylation, and thus support EGF-stimulated cell proliferation and cell-cell adhesion in *MGAT1* mutant cells.

## Discussion

To decipher the role of oligomannose N-glycans in NB growth and progression, our laboratory has engineered N-glycosylation mutant NB cell lines. Herein the expression of the *MGAT1* gene was reduced in two cell colonies of the human NB cell line, BE(2)-M17, to further ascertain the influential role of oligomannose N-glycans on cancerous cellular properties, including their impact on the EGFR signaling pathway. We demonstrated that the introduction of indels in *MGAT1* resulted in markedly reduced GnT-I activity as levels of oligomannose type N-glycans were escalated, while the higher order glycan types, complex and hybrid, plummeted. Previous studies have reported three cell types in NB cell lines, including I-, S-, and N-types [36–38]. All three types were observed in the parental, BE(2)-M17, and the *MGAT1* mutants, Trunc and ∆45 cell lines; however, the percentages of S-type and N-type were increased and decreased, respectively, in the *MGAT1* mutant cell lines relative to the parental cell line. Anchorage-independent growth and the proliferation rate at sub-confluency in the *MGAT1* mutant cells were slower than BE(2)-M17. However, when cells were cultured at and above confluency the *MGAT1* mutant cells proliferated at a faster rate. Further cell spheroid area was markedly increased in the *MGAT1* mutant lines after 1 d incubation due to heightened cell proliferation. Cell-cell adhesion and cell invasiveness were increased in the *MGAT1* mutant lines compared to BE(2)-M17. Following EGF stimulation, *MGAT1* mutant lines had increased levels of P-EGFR (Y1068) which was accompanied by enhanced cell proliferation and cell-cell adhesion. Novel findings were that increased oligomannose type N-glycans in NB cells maintained relatively higher proliferation rates at higher cell densities, and higher EGF stimulation leading to increased cell growth and adhesion. Taken together, raised oligomannose type N-glycans accompanied with decreased levels of higher order types of N-glycans in the tumor microenvironment markedly enhanced NB cell growth and invasiveness, and therefore oligomannose N-glycans are major determinants in the development and progression of NB.

Past studies have shown that S-type cells express higher levels of vimentin while N-type cells express higher amounts of neurofilament [36–38]. Here all cell lines consisted of all three cell types; however, BE(2)-M17 had lower levels of S-type and was observed to express slightly less vimentin than the *MGAT1* mutant lines. Further neurofilament was not detected in any of the cell lines, including BE(2)-M17 which expressed more N-type cells by at least 2-fold. Previously we showed that neurofilament was very abundant in rat NB cells which predominantly expressed N-type [26]. Thus, lowered GnT-I activity and raised levels of oligomannose N-glycans in the *MGAT1* mutant cell lines encouraged S-type cells and slightly raised levels of vimentin.

A remarkable finding was the change in cell proliferation rates between the BE(2)-M17 and *MGAT1* mutant cell lines with increased cell density. Cell proliferation rates were slower at sub-confluency but faster on confluent to over-confluent dishes in *MGAT1* mutant cell lines relative to the parental cell line. Both I- and N-type cells grow more rapidly than S-type [36,37] which is supported by our finding that the parental cell line proliferates faster than the *MGAT1* mutant cell lines when examining cell proliferation at sub-confluency. Similar findings were also observed in rat and human NB cell lines [19,24]. In contrast, when cell proliferation was tested at and above confluency the mutant cell lines were faster than the BE(2)-M17 cell line. It has been reported that S-type cells adhered more strongly to the substratum than I- and N-types [36–38]. As such, a contributing factor for the change in proliferation rates at high cell densities could be that both Trunc and ∆45 cell lines express higher levels of S-type cells so these cell lines can adhere to the substratum better and pack tighter and thereby continue to proliferate at a high rates at high cell densities. The importance of cell adhesion in increasing NB cell proliferation is also supported by our observation that cell spheroids of the *MGAT1* mutant lines were faster than BE(2)-M17, along with our finding that cell-cell adherence was stronger when oligomannose N-glycans replaced higher order N-glycans. In general, cancer cells lack contact inhibition of proliferation [39]. As expected, our current results support this concept; however, they revealed that contact inhibition of proliferation has a lesser effect when complex/hybrid N-glycans were substituted with oligomannose N-glycans in the cellular microenvironment. Hence, the replacement of complex/hybrid type N-glycans with oligomannose type N-glycans in NB cells maintains proliferation rates when cells are at high densities by strengthening cell adherence and reducing contact inhibition of proliferation.

Increased levels of oligomannose N-glycans in the tumor microenvironment have a tremendous impact on NB cell invasiveness. Our past studies have implicated oligomannose N-glycans as promoters of cell invasiveness in rat [24] and human [19,33] NB cell lines. Oligomannose N-glycans are abundant in numerous cancer cell lines [23], including NB cell lines [24,25]. Moreover, these N-glycans have been linked to the progression of NB, breast, ovarian, liver, and prostate cancer [23,24,27,28,30,40]. Here we showed that cell invasiveness was enhanced in two independent cell colonies with introduction of indels in the *MGAT1* gene. Accordingly, our current study expands the role of oligomannose N-glycans to the promotion of NB cell invasiveness and thereby the progression of NB.

Replacement of higher order N-glycans with oligomannose N-glycans of EGFR promote EGFR activation and downstream signaling which modulate cell growth and adhesion. N-glycosylated EGFR is a substrate of GnT-II [14,41–43], and it is abundant in NB cells and tissue [8,9]. A recent report showed that complex N-glycans were attached to EGFR in a human NB cell line, BE(2)-C, while oligomannosylated EGFR was observed in the *MGAT1* mutant BE(2)-C cell line, referred to as BE(2)-C(-*MGAT1*) [19]. Based on the electrophoretic migration of EGFR observed in BE(2)-M17 and the *MGAT1* mutant cell lines along with those previously reported for BE(2)-C and BE(2)-C(-*MGAT1*) [19], our results support that the BE(2)-M17 cell line expressed glycosylated EGFR with enriched complex N-glycans, and the *MGAT1* mutant cell lines had EGFR with mainly oligomannose N-glycans. Further EGFR signaling enhanced cell proliferation [19] and adhesion, when EGFR was oligomannosylated. Taken together, EGF-stimulated cell proliferation and cell adhesion were markedly enhanced when N-glycosylated EGFR had oligomannose N-glycans, instead of complex N-glycans.

## Conclusions

Herein, we expand our studies to ascertain the role of oligomannose N-glycans in the development and progression of NB. A novel assignment of oligomannose N-glycans in NB cells is the perpetuation of high proliferation rates at high cell densities and thereby weakened contact inhibition of cell proliferation. Secondly, higher levels of oligomannose N-glycans favored S-type cells. In expansion of our prior study [19], cells expressing oligomannosylated EGFR were much more sensitive to EGF stimulation as autophosphorylation, cell proliferation and cell adhesion were heightened. Finally, the establishment that raised oligomannose N-glycans promotes NB cell invasiveness in yet another NB cell line. Thus, the increased expression of oligomannose N-glycans in NB cells promotes NB growth and invasiveness, and furthermore, intensifies EGF stimulated proliferation and adhesion.

## Supporting information

S1 Fig Western blot of the phosphorylation of Y1068 in EGFR from whole cell lysates of BE(2)-M17, Trunc and ∆45 with (+) and without (−) 20 ng/mL EGF. P-EGFR was undetected in absence of EGF. Originals of Western, and lectin blots, plus Comassie blue stained gels. Supplementary Group Data.
(ZIP)

## References

[1] Zhou X, Wang X, Li N, Guo Y, Yang X, Lei Y. Therapy resistance in neuroblastoma: mechanisms and reversal strategies. Front Pharmacol. 2023;14:1114295. doi: 10.3389/fphar.2023.1114295 36874032 PMC9978534

[2] Gerges A, Canning U. Neuroblastoma and its target therapies: a medicinal chemistry review. ChemMedChem. 2024;19(9):e202300535. doi: 10.1002/cmdc.202300535 38340043

[3] Sainero-Alcolado L, Sjöberg Bexelius T, Santopolo G, Yuan Y, Liaño-Pons J, Arsenian-Henriksson M. Defining neuroblastoma: from origin to precision medicine. Neuro Oncol. 2024;26(12):2174–92. doi: 10.1093/neuonc/noae152 39101440 PMC11630532

[4] Ho W-L, Hsu W-M, Huang M-C, Kadomatsu K, Nakagawara A. Protein glycosylation in cancers and its potential therapeutic applications in neuroblastoma. J Hematol Oncol. 2016;9(1):100. doi: 10.1186/s13045-016-0334-6 27686492 PMC5041531

[5] Stanley P, Moremen KW, Lewis NE, Taniguchi N, Aebi M. N-Glycans. In: Varki A, Cummings RD, Esko JD, Stanley P, Hart GW, Aebi M, editors. Essentials of glycobiology. Cold Spring Harbor (NY): Cold Spring Harbor Laboratory Press; 2022. 35536922

[6] Nicholson RI, Gee JM, Harper ME. EGFR and cancer prognosis. Eur J Cancer. 2001;37 Suppl 4:S9-15. doi: 10.1016/s0959-8049(01)00231-3 11597399

[7] Kang X, Li R, Li X, Xu X. EGFR mutations and abnormal trafficking in cancers. Mol Biol Rep. 2024;51(1):924. doi: 10.1007/s11033-024-09865-z 39167290

[8] Ho R, Minturn JE, Hishiki T, Zhao H, Wang Q, Cnaan A, et al. Proliferation of human neuroblastomas mediated by the epidermal growth factor receptor. Cancer Res. 2005;65(21):9868–75. doi: 10.1158/0008-5472.CAN-04-2426 16267010

[9] Zheng C, Shen R, Li K, Zheng N, Zong Y, Ye D, et al. Epidermal growth factor receptor is overexpressed in neuroblastoma tissues and cells. Acta Biochim Biophys Sin (Shanghai). 2016;48(8):762–7. doi: 10.1093/abbs/gmw064 27353319

[10] Keller J, Nimnual AS, Varghese MS, VanHeyst KA, Hayman MJ, Chan EL. A novel EGFR extracellular domain mutant, EGFRΔ768, possesses distinct biological and biochemical properties in neuroblastoma. Mol Cancer Res. 2016;14(8):740–52. doi: 10.1158/1541-7786.MCR-15-0477 27216155 PMC4987210

[11] Stafman LL, Beierle EA. Cell proliferation in neuroblastoma. Cancers (Basel). 2016;8(1):13. doi: 10.3390/cancers8010013 26771642 PMC4728460

[12] Takahashi M, Hasegawa Y, Gao C, Kuroki Y, Taniguchi N. N-glycans of growth factor receptors: their role in receptor function and disease implications. Clin Sci (Lond). 2016;130(20):1781–92. doi: 10.1042/CS20160273 27612953

[13] Ling Y-H, Li T, Perez-Soler R, Haigentz M Jr. Activation of ER stress and inhibition of EGFR N-glycosylation by tunicamycin enhances susceptibility of human non-small cell lung cancer cells to erlotinib. Cancer Chemother Pharmacol. 2009;64(3):539–48. doi: 10.1007/s00280-008-0902-8 19130057

[14] Soderquist AM, Carpenter G. Glycosylation of the epidermal growth factor receptor in A-431 cells. The contribution of carbohydrate to receptor function. J Biol Chem. 1984;259(20):12586–94. doi: 10.1016/s0021-9258(18)90787-8 6092339

[15] Wang Y, Zhang L, He Z, Deng J, Zhang Z, Liu L, et al. Tunicamycin induces ER stress and inhibits tumorigenesis of head and neck cancer cells by inhibiting N-glycosylation. Am J Transl Res. 2020;12(2):541–50. 32194902 PMC7061826

[16] Slieker LJ, Lane MD. Post-translational processing of the epidermal growth factor receptor. Glycosylation-dependent acquisition of ligand-binding capacity. J Biol Chem. 1985;260(2):687–90. doi: 10.1016/s0021-9258(20)71149-x 2981842

[17] Takahashi M, Hasegawa Y, Maeda K, Kitano M, Taniguchi N. Role of glycosyltransferases in carcinogenesis; growth factor signaling and EMT/MET programs. Glycoconj J. 2022;39(2):167–76. doi: 10.1007/s10719-022-10041-3 35089466 PMC8795723

[18] Gabius H-J, van de Wouwer M, André S, Villalobo A. Down-regulation of the epidermal growth factor receptor by altering N-glycosylation: emerging role of β1,4-galactosyltransferases. Anticancer Res. 2012;32(5):1565–72. 22593433

[19] Burch AP, Kristen Hall M, Wease D, Schwalbe RA. Reduction of N-acetylglucosaminyltransferase-I activity promotes neuroblastoma invasiveness and EGF-stimulated proliferation in vitro. Int J Transl Med (Basel). 2024;4(3):519–38. doi: 10.3390/ijtm4030035 39742082 PMC11687401

[20] Kawashima N, Yoon S-J, Itoh K, Nakayama K. Tyrosine kinase activity of epidermal growth factor receptor is regulated by GM3 binding through carbohydrate to carbohydrate interactions. J Biol Chem. 2009;284(10):6147–55. doi: 10.1074/jbc.M808171200 19124464

[21] Liu Y-C, Yen H-Y, Chen C-Y, Chen C-H, Cheng P-F, Juan Y-H, et al. Sialylation and fucosylation of epidermal growth factor receptor suppress its dimerization and activation in lung cancer cells. Proc Natl Acad Sci U S A. 2011;108(28):11332–7. doi: 10.1073/pnas.1107385108 21709263 PMC3136320

[22] Partridge EA, Le Roy C, Di Guglielmo GM, Pawling J, Cheung P, Granovsky M, et al. Regulation of cytokine receptors by Golgi N-glycan processing and endocytosis. Science. 2004;306(5693):120–4. doi: 10.1126/science.1102109 15459394

[23] Chatterjee S, Ugonotti J, Lee LY, Everest-Dass A, Kawahara R, Thaysen-Andersen M. Trends in oligomannosylation and α1,2-mannosidase expression in human cancers. Oncotarget. 2021;12(21):2188–205. doi: 10.18632/oncotarget.28064 34676051 PMC8522845

[24] Hall MK, Burch AP, Schwalbe RA. Functional analysis of N-acetylglucosaminyltransferase-I knockdown in 2D and 3D neuroblastoma cell cultures. PLoS One. 2021;16(11):e0259743. doi: 10.1371/journal.pone.0259743 34748597 PMC8575246

[25] Hall MK, Hatchett CJ, Shalygin S, Azadi P, Schwalbe RA. Reduction in N-acetylglucosaminyltransferase-I activity decreases survivability and delays development of zebrafish. Curr Issues Mol Biol. 2023;45(11):9165–80. doi: 10.3390/cimb45110575 37998752 PMC10669939

[26] Hall MK, Shajahan A, Hatchett CJ, Burch AP, Azadi P, Schwalbe RA. Kv3-expressing cells present more elaborate N-glycans with changes in cytoskeletal proteins, neurite structure and cell migration. COJ Biomed Sci Res. 2023;2(3):540. doi: 10.31031/cojbsr.2023.02.000540 39736999 PMC11684427

[27] Briggs MT, Condina MR, Ho YY, Everest-Dass AV, Mittal P, Kaur G, et al. MALDI mass spectrometry imaging of early- and late-stage serous ovarian cancer tissue reveals stage-specific N-glycans. Proteomics. 2019;19(21–22):e1800482. doi: 10.1002/pmic.201800482 31364262

[28] Gilgunn S, Murphy K, Stöckmann H, Conroy PJ, Murphy TB, Watson RW. Glycosylation in indolent, significant and aggressive prostate cancer by automated high-throughput N-glycan profiling. Int J Mol Sci. 2020;21(23). doi: 10.3390/ijms21239233 33287410 PMC7730228

[29] Li Q, Li G, Zhou Y, Zhang X, Sun M, Jiang H, et al. Comprehensive N-glycome profiling of cells and tissues for breast cancer diagnosis. J Proteome Res. 2019;18(6):2559–70. doi: 10.1021/acs.jproteome.9b00073 30889355

[30] Takayama H, Ohta M, Iwashita Y, Uchida H, Shitomi Y, Yada K, et al. Altered glycosylation associated with dedifferentiation of hepatocellular carcinoma: a lectin microarray-based study. BMC Cancer. 2020;20(1):192. doi: 10.1186/s12885-020-6699-5 32143591 PMC7060603

[31] Hall MK, Shajahan A, Burch AP, Hatchett CJ, Azadi P, Schwalbe RA. Limited N-glycan processing impacts chaperone expression patterns, cell growth and cell invasiveness in neuroblastoma. Biology. 2023;12(2):293. doi: 10.3390/biology12020293 36829569 PMC9953357

[32] Ran FA, Hsu PD, Wright J, Agarwala V, Scott DA, Zhang F. Genome engineering using the CRISPR-Cas9 system. Nat Protoc. 2013;8(11):2281–308. doi: 10.1038/nprot.2013.143 24157548 PMC3969860

[33] Burch AP, Hall MK, Schwalbe RA. Neuroblastoma cell growth and invasiveness is modulated by the activity of N-acetylglucosaminyltransferase-III. PLoS One. 2026;21(6):e0350822. doi: 10.1371/journal.pone.0350822 42258445 PMC13245742

[34] Kumar R, Yang J, Larsen RD, Stanley P. Cloning and expression of N-acetylglucosaminyltransferase I, the medial Golgi transferase that initiates complex N-linked carbohydrate formation. Proc Natl Acad Sci U S A. 1990;87(24):9948–52. doi: 10.1073/pnas.87.24.9948 1702225 PMC55291

[35] Bojar D, Meche L, Meng G, Eng W, Smith DF, Cummings RD, et al. A useful guide to lectin binding: machine-learning directed annotation of 57 unique lectin specificities. ACS Chem Biol. 2022;17(11):2993–3012. doi: 10.1021/acschembio.1c00689 35084820 PMC9679999

[36] Bell N, Hann V, Redfern CPF, Cheek TR. Store-operated Ca(2+) entry in proliferating and retinoic acid-differentiated N- and S-type neuroblastoma cells. Biochim Biophys Acta. 2013;1833(3):643–51. doi: 10.1016/j.bbamcr.2012.11.025 23220046 PMC3776921

[37] Ross RA, Biedler JL, Spengler BA. A role for distinct cell types in determining malignancy in human neuroblastoma cell lines and tumors. Cancer Lett. 2003;197(1–2):35–9. doi: 10.1016/s0304-3835(03)00079-x 12880957

[38] Walton JD, Kattan DR, Thomas SK, Spengler BA, Guo H-F, Biedler JL, et al. Characteristics of stem cells from human neuroblastoma cell lines and in tumors. Neoplasia. 2004;6(6):838–45. doi: 10.1593/neo.04310 15720811 PMC1531688

[39] Grimes DR, Fletcher AG. Close encounters of the cell kind: the impact of contact inhibition on tumour growth and cancer models. Bull Math Biol. 2020;82(2):20. doi: 10.1007/s11538-019-00677-y 31970500 PMC6976547

[40] Li D, Mukhopadhyay S. Functional analyses of the UDP-galactose transporter SLC35A2 using the binding of bacterial Shiga toxins as a novel activity assay. Glycobiology. 2019;29(6):490–503. doi: 10.1093/glycob/cwz016 30834435 PMC6521944

[41] Cummings RD, Soderquist AM, Carpenter G. The oligosaccharide moieties of the epidermal growth factor receptor in A-431 cells. Presence of complex-type N-linked chains that contain terminal N-acetylgalactosamine residues. J Biol Chem. 1985;260(22):11944–52. doi: 10.1016/s0021-9258(17)38969-x 2995354

[42] Takahashi M, Yokoe S, Asahi M, Lee SH, Li W, Osumi D, et al. N-glycan of ErbB family plays a crucial role in dimer formation and tumor promotion. Biochim Biophys Acta. 2008;1780(3):520–4. doi: 10.1016/j.bbagen.2007.10.019 18036567

[43] Whitson KB, Whitson SR, Red-Brewer ML, McCoy AJ, Vitali AA, Walker F, et al. Functional effects of glycosylation at Asn-579 of the epidermal growth factor receptor. Biochemistry. 2005;44(45):14920–31. doi: 10.1021/bi050751j 16274239

