# Supplementary material for: Mutations in the N-glycan branching enzyme MGAT1 promote invasiveness and EGF-stimulated proliferation and adhesion in neuroblastoma cells
Source: PLoS One. 2026 Sep 10;21(9):e0358077. doi: 10.1371/journal.pone.0358077 (PMC13561367; doi:10.1371/journal.pone.0358077)
Supplement: Supplementary file 1 [file pone.0358077.s001.zip › Supplementary Group Data_260722.pdf]

Fig 9 (data group)	**Fig 9A**	**Be(2)-M17**	**Trunc**	**Δ45**	**Fig 9C**
		0.94737	1.08571	1.27451	
		0.7193	1.4	1.54902	
EGF-stimulated cell proliferation		0.85965	1.05714	1.19608	
and cell adhesion		0.96491	1.54286	1.41176	
		0.87719	1.17143	1.41176	

Be(2)-M17	Trunc	Δ45
1.14667	1.74647	1.66365
0.84388	1.04552	1.66365
1.23431	2.05571	1.95714
0.9973	1.43927	0.74868
0.65519	1.2034	2.51698
1.33114	1.44293	1.12218
0.75679	1.38247	0.94253
1.12738	0.97174	1.10757
0.98866	1.10122	1.1457
0.82974	1.08016	0.76357
0.89514	1.1606	0.63206
0.8279	1.33967	1.07501
1.62511	2.37663	1.46911
1.9099	1.28546	1.0892
0.98131	1.01834	2.34553
1.36605	1.75476	1.4467
1.27933	1.64063	1.45157
0.96312	0.84959	1.25703
0.84058	1.46522	1.07041
1.09945	1.2	0.90287
0.71435	1.05136	1.54926
1.23358	1.11685	1.13582
1.10368	0.93329	1.1297
1.01567	1.22446	0.77609
1.07851	1.56916	0.58113
0.93538	2.68859	0.7292
1.58138	1.48927	0.613
1.34088	0.98764	1.33287
0.86262	1.05543	0.93126
1.04415	0.9841	0.93348
0.69267	0.73492	1.89619
0.75349	0.83179	1.20443
0.88706	1.58804	0.67659
0.97801	0.89796	1.31547
1.00208	0.92174	3.06777
0.969	1.91114	1.54314
0.68661	1.31073	1.06596
0.76028	0.76807	1.188
0.84572	1.29633	1.25118
0.9916	1.35177	1.52394
0.98719	1.79511	0.81005
0.8527	2.43696	0.51851
1.32104	1.34293	0.82814

1.26445	1.31549	1.06415
0.97819	0.86793	1.28222
1.15347	0.87052	0.79432
0.86722	1.32323	0.79627
0.72831	1.4269	1.24325
0.83708	1.51073	0.79975
1.0741	1.78179	1.38394
0.77131	1.11685	1.19621
1.10442	1.06223	1.50306
1.04286	1.24361	2.043
0.88118	0.82446	0.75132
0.68973	0.72242	0.8781
0.9769	1.98533	0.85834
0.91499	0.90842	1.7452
0.91554	1.55965	1.07362
0.59805	0.89049	1.05831
0.68973	2.04171	1.25494
0.89404	0.77486	1.28681
0.81724	1.08451	1.53896
0.84848	1.7481	1.34734
1.14208	1.28967	1.71681
0.86832	0.70516	0.90802
1.2108	1.44606	1.64208
1.06142	1.91467	1.21806
0.97066	1.54049	1.17019
0.74559	1.37446	1.18272
0.80952	1.49918	1.69051
0.87346	1.81916	1.05288
1.07961	1.03125	0.99457
1.04139	1.91291	1.06624
0.71894	1.55476	1.03632
0.73511	1.62296	1.14793
1.02908	0.72446	1.11745
0.75771	0.95353	1.02254
1.24001	2.1053	1.53841
0.97268	1.5125	1.35736
1.07943	1.42717	0.97342
1.01659	2.33614	1.355
0.62726	1.28247	1.21403
0.78141	2.32799	0.66727
0.77939	1.54606	1.11522
0.74908	1.97976	0.79738
0.91444	1.37582	1.00612
0.94971	1.41671	0.71667

0.79519	1.5303	0.95825
0.66732	1.65883	1.34748
0.73916	0.91997	1.06276
1.01898	0.87826	0.68244
1.16541	1.05584	1.21598
0.69745	1.21916	0.66671
0.92987	1.14511	0.71848
1.88068	2.05041	0.81116
0.98297	0.92201	1.1681
1.02835	1.20448	0.78556
0.93189	1.68478	0.8536
0.86997	2.17242	0.68035
1.28282	1.31264	0.66504
0.82275	1.12228	1.10674
1.54151	1.10734	0.63791
0.89257	1.29565	1.0167
0.84315	0.62296	0.62163
0.92179	0.6909	1.03896
0.74099	0.8519	0.98066
0.75679	0.84402	1.13178
0.75257	0.66834	1.5359
0.96166	1.00109	0.84205
0.51114	0.9856	0.68508
0.81412	1.01957	1.18703
0.90286	1.19198	1.58127
0.96423	1.26916	0.78695
0.88982	1.11033	1.22864
0.92932	0.77948	1.00487
0.94273	1.10285	0.69274
0.90874	0.87323	1.32619
1.64238	1.09864	1.13192
0.65207	1.29307	1.2054
0.71123	0.89579	1.03549
0.89276	1.00707	1.19914
0.69138	1.01128	1.72627
0.84499	1.29375	0.73365
0.77553	1.49918	0.96827
0.77076	1.03084	0.82856
0.98958	1.25299	1.51517
0.67687	1.08071	1.24102
0.77553	1.01427	1.0622
1.00336	1.45489	1.45964
1.23174	0.89334	1.1553
0.77315	1.11467	1.18286

1.18673	1.05978	0.89674
0.95118	0.89375	1.55803
0.73934	1.70802	0.88575
0.7827	0.92418	0.8966
1.47261	0.58764	1.269
1.32857	1.09742	1.22099
1.12426	1.02976	0.84331
0.74926	1.35462	1.5462
0.7274	0.6356	1.08141
	1.02514	0.93905
	1.15951	1.32814
	2.39253	0.99499
	1.93736	1.33384
	1.15326	1.27888
	1.03166	2.73574
	1.00068	1.85903
	0.84429	0.85249
	0.94592	1.52992
	1.55774	1.46813
	1.91277	0.78291
	1.67609	2.23963
	1.17296	1.66991
	1.5856	0.9147
	1.49918	1.01531
	1.43302	0.91428
	1.59511	1.75035
	0.91019	1.11815
	1.18832	1.22864
	1.51997	1.04411
	1.83886	
	1.16168	
	1.94049	
	1.66073	
	1.37188	
	0.97378	
	1.70679	
	1.13152	
	1.37337	
	1.15408	
	1.09484	
	1.3288	
	1.17921	
	1.18179	
	1.88207	
