# Supplementary material for: Mutations in the N-glycan branching enzyme MGAT1 promote invasiveness and EGF-stimulated proliferation and adhesion in neuroblastoma cells
Source: PLoS One. 2026 Sep 10;21(9):e0358077. doi: 10.1371/journal.pone.0358077 (PMC13561367; doi:10.1371/journal.pone.0358077)
Supplement: Supplementary file 1 [file pone.0358077.s001.zip › Fig S1.pdf]

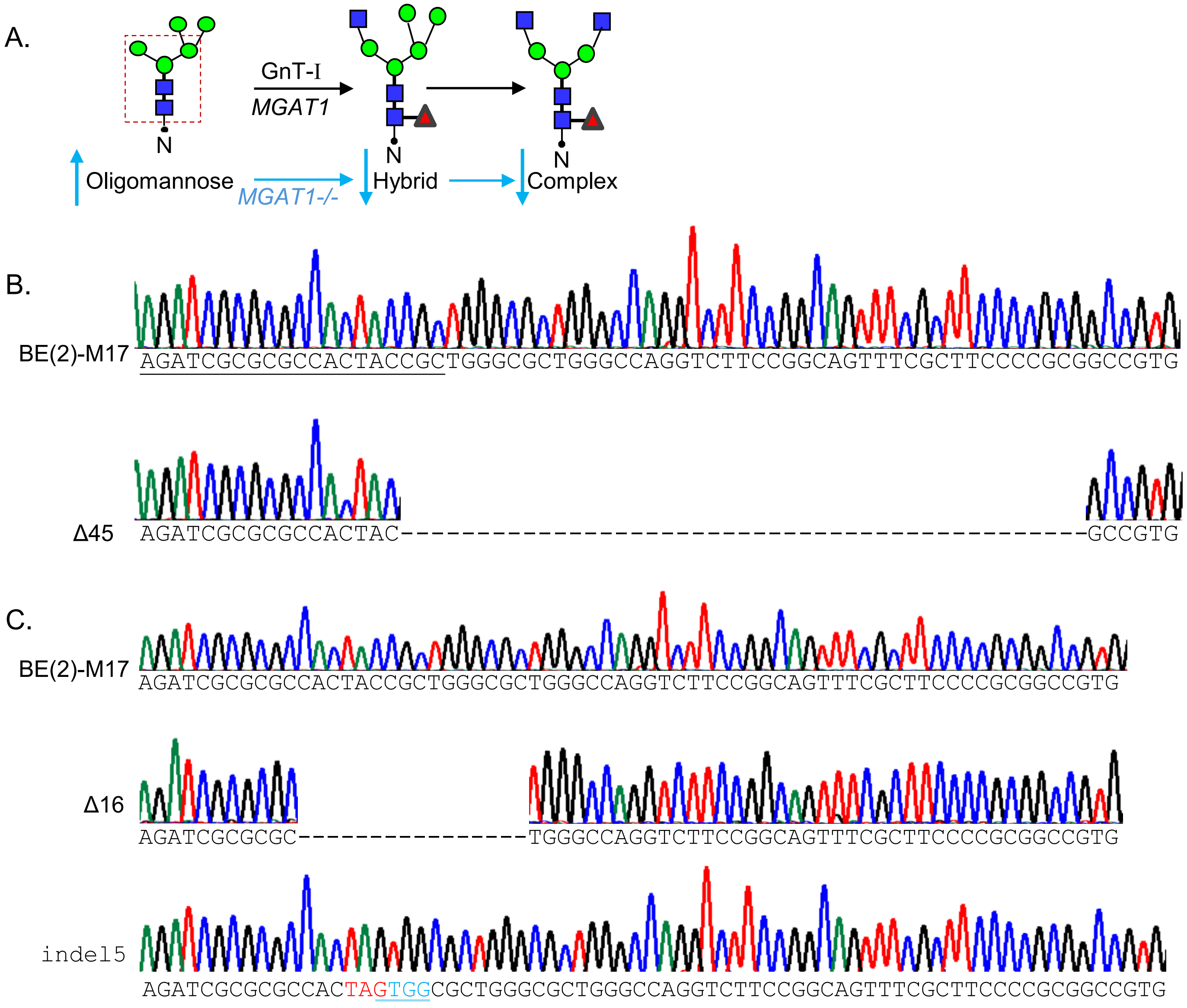
A.
GnT-I
MGAT1
N
Oligomannose
MGAT1-/-
Hybrid
Complex
B.
BE(2)-M17
AGATCGCGCGCCACTACCGCTGGGCGCTGGGCCAGGTCTTCCGGCAGTTTCGCTTCCCCGCGGCCGTG
Δ45
AGATCGCGCGCCACTAC---GCCGTG
C.
BE(2)-M17
AGATCGCGCGCCACTACCGCTGGGCGCTGGGCCAGGTCTTCCGGCAGTTTCGCTTCCCCGCGGCCGTG
Δ16
AGATCGCGCGC----------------TGGGCCAGGTCTTCCGGCAGTTTCGCTTCCCCGCGGCCGTG
indel5
AGATCGCGCGCCACTAGTGGCGCTGGGCGCTGGGCCAGGTCTTCCGGCAGTTTCGCTTCCCCGCGGCCGTG

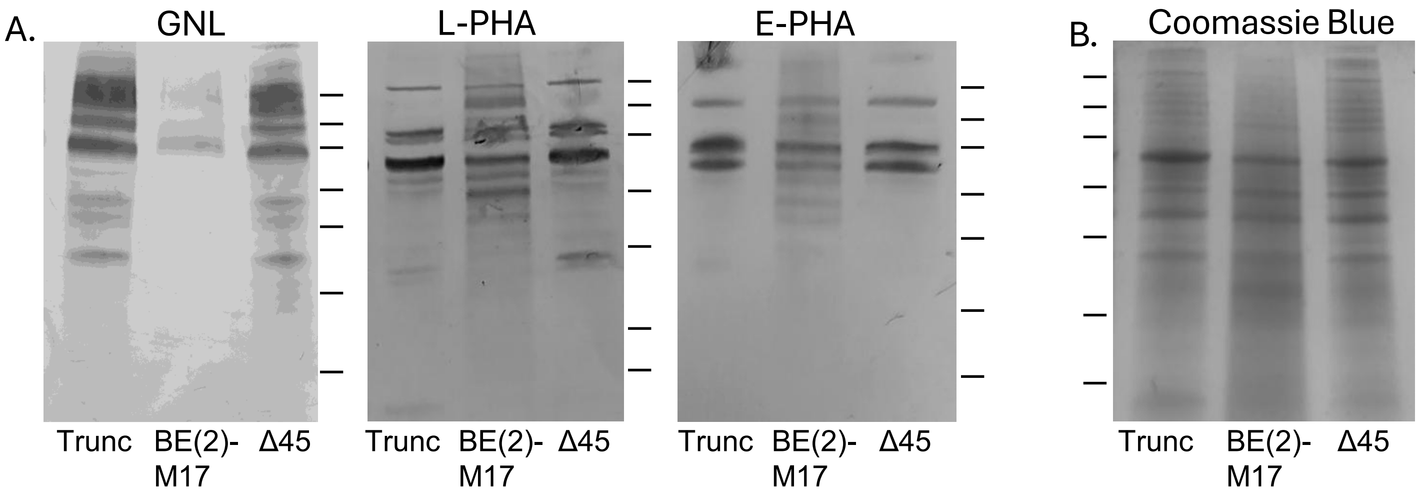

C.

Lectin blot to shown expression of GnT-I increases GNL binding

Figure 2: Lectin binding of glycoproteins in parental and glycosylation mutant cell lines. A) Whole cell lysates from Trunc, BE(2)-M17 and ∆45 cell lines. Separated N-glycosylated proteins from the various cell lines were probed with GNL, L-PHA, and E-PHA lectins. B) Coomassie blue-stained gel of total protein from the various cell lines. C) GNL probed blots of N-glycosylated proteins from whole cell lysates of BE(2)-M17, Trunc and ∆45 cells without (-) and with (+) expression of heterologous GnT-I, D) along with Coomassie blue-stained gel of the samples. Lines adjacent to membranes and gel denote molecular weights markers of 250, 150, 100, 75, 50, 37 and 25 kDa.

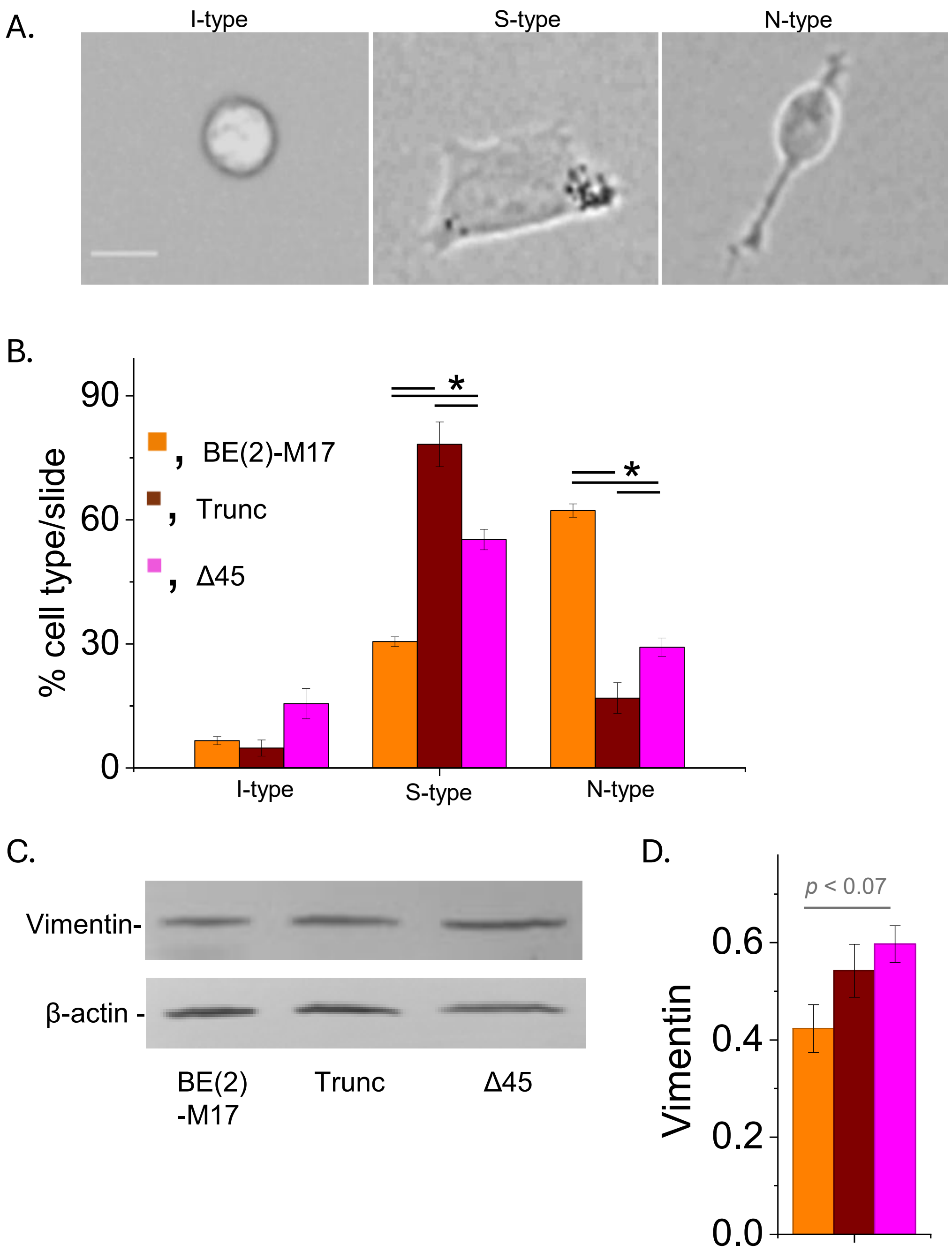

Figure 3: Variation of cell types between BE(2)-M17, Trunc, and Δ45 cell lines. A) I-type, S-type, and N-type cells were observed in all three cell lines. B) Percent of cell type per slide for BE(2)-M17 (*n*=3), Trunc (*n*=4), and Δ45 (*n*=4) cell lines. C) Western blot of vimentin, and D) quantification of vimentin immunoband with respect to β-actin from BE(2)-M17 (*n*=9), Trunc (*n*=6), and Δ45 (*n*=6), where *n* denotes number of Western blots. Two biological samples were used. Scale bar is 10 μm. Numerical values are represented as mean ± SEM. One-way ANOVA with a post hoc Holm-Bonferroni was used to compare means, where *p<0.05, and as indicated.

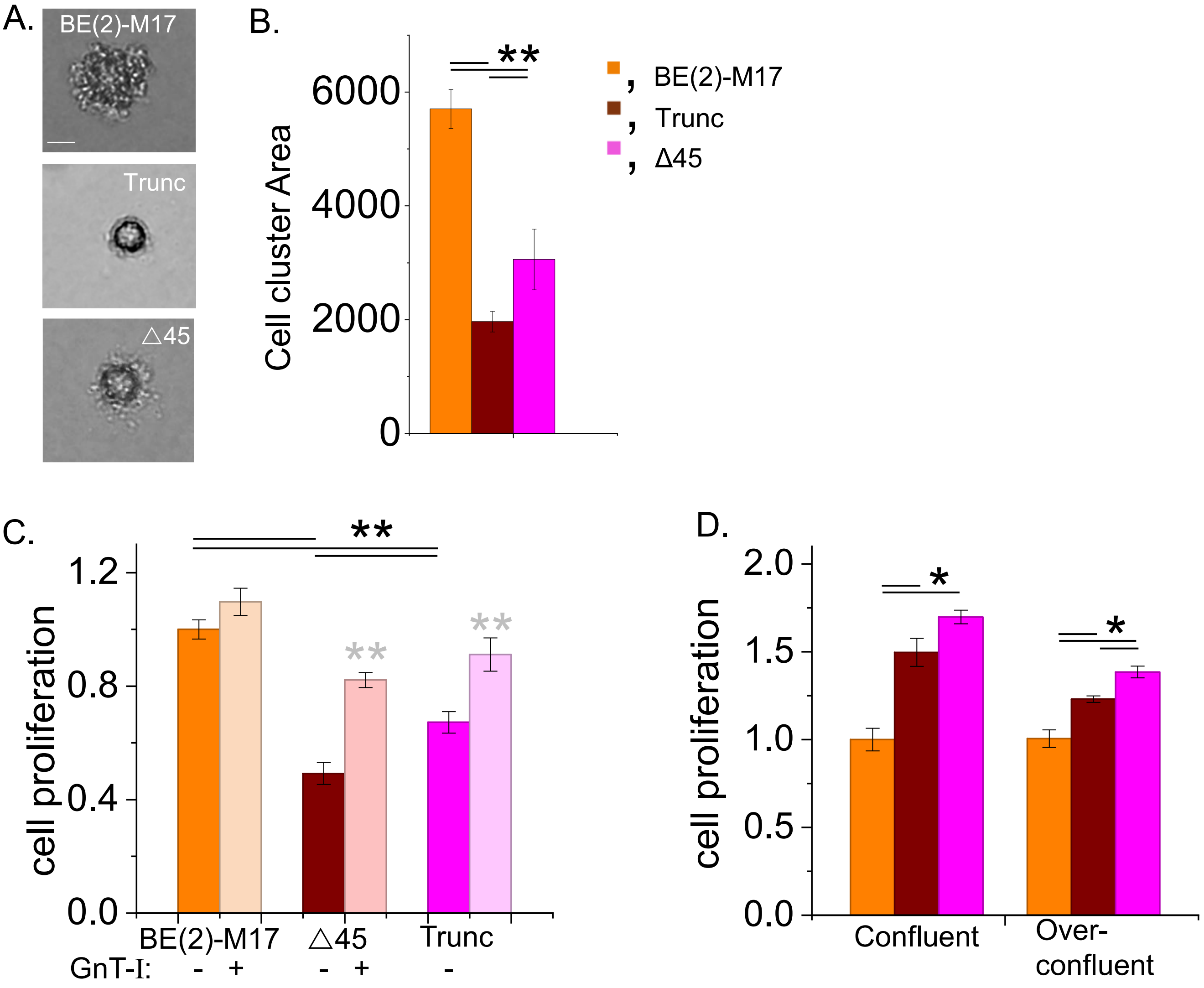

Figure 4: Deficient GnT-I cell lines had retarded non-adhered and adhered cell growth. A) Typical DIC images of cell colonies from anchorage independent growth of parental and N-glycosylated mutant cell lines. B) Cell growth was monitored by quantification of the area of cell colonies from BE(2)-M17 (*n*=144), Trunc (*n*=105), and Δ45 (*n*=63) following 13 days of growth where *n* is the number of cell clusters. C) Sub-confluent cell monolayers of BE(2)-M17 (*n*=13), Trunc (*n*=9), and Δ45 (*n*=9), along with BE(2)-M17 (*n*=4), Trunc (*n*=4), and Δ45 (*n*=4) ectopically expressing GnT-I were analyzed via BrdU cell proliferation assay. The fainter colors of BE(2)-M17 (■), Δ45 (■), and Trunc (■) represent cell lines overexpressing Mgat1. D) Adhered cells at confluency and over-confluency were monitored using the BrdU cell proliferation assay in (BE(2)-M17, Trunc, and Δ45 (*n*=4). *n* denotes number of replicates. Scale bar is 5 µm. Data are presented as mean ± SEM. One-way ANOVA with a post hoc Holm-Bonferroni was used when comparing more than two samples, where *p<0.05 and **p<0.01.. Student T-test was used to compare each cell line without and with GnT-I in panel C, where **p< 0.01.

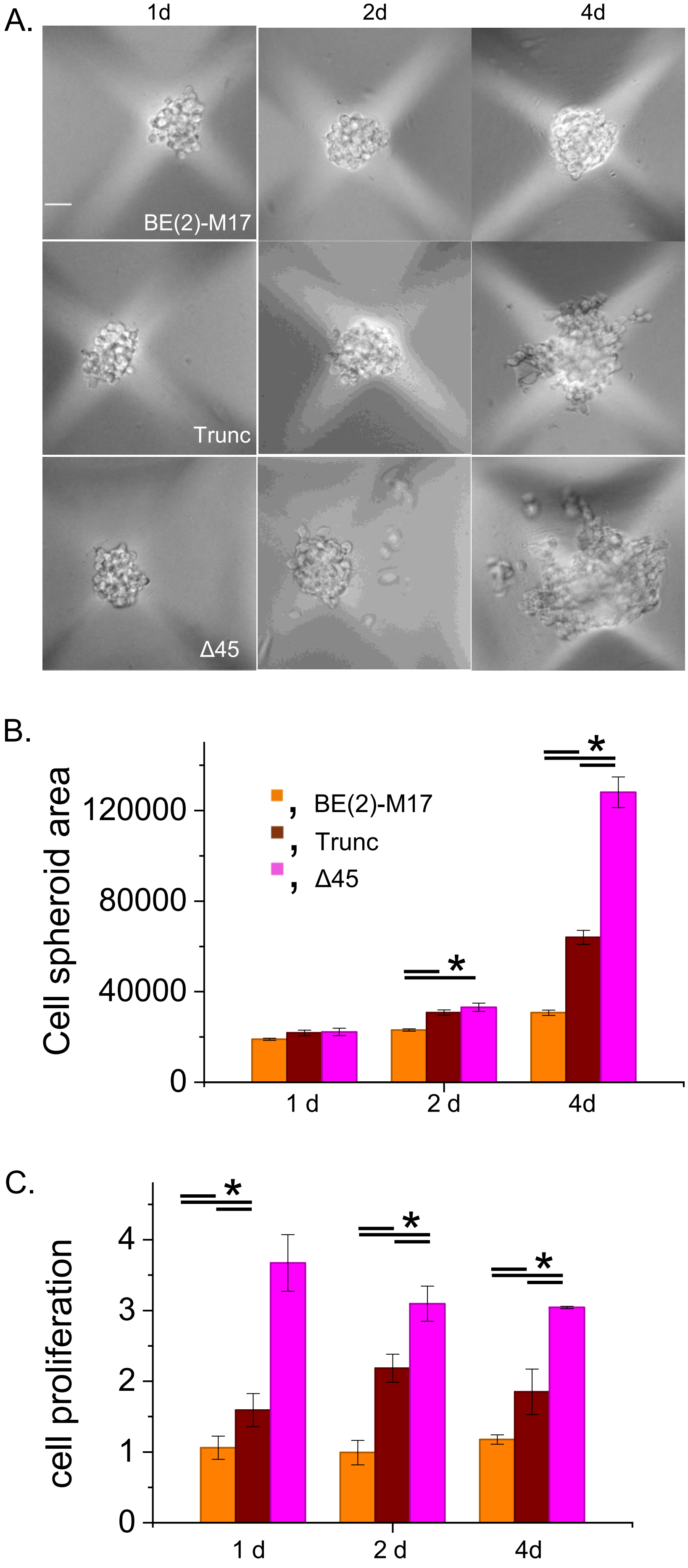

Figure 5: Cell spheroid f
parental and mutant cell
micrographs of spheroids
Scale bar is 25 µm. B) Qu
at 1,2, and 4 days from BE
Trunc (*n*=79, 120, 61), and
lines. *n* denotes number o
proliferation of 1, 2 and 4 d
each sample, as indicated
replicates. Data are repres
One-way ANOVA with a po
used to compare means, w

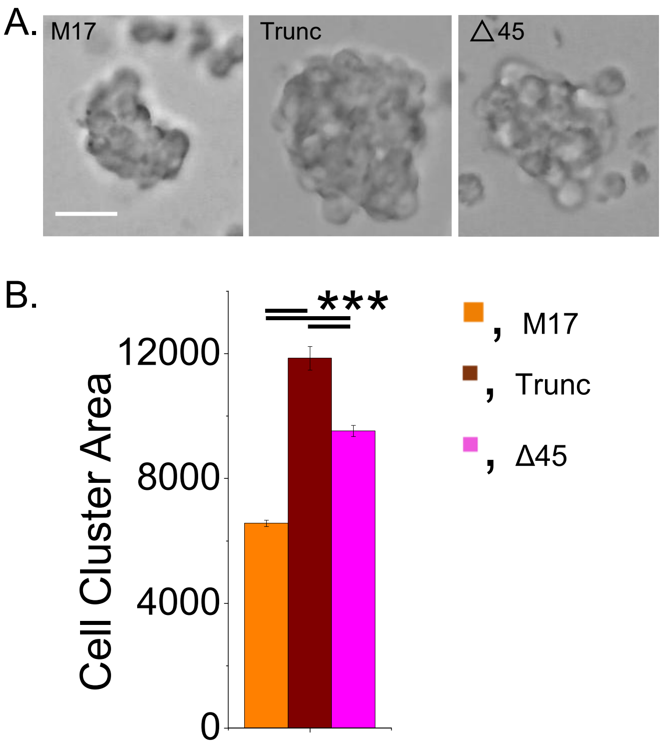

Figure 6: Lowered GnT-I activity enhances cell-cell adhesion. A) Illustrative microscopic images of intact cell clusters of BE(2)-M17, Trunc, and Δ45 after mechanical dissociation of a cell monolayer via pipetting. B) The area of cell clusters from BE(2)-M17 (*n*=508), Trunc (*n*=292), and Δ45 (*n*=594). *n* denotes the number cell clusters. Scale bar is 25 µm. Quantifications are signified as mean ± SEM. One-way ANOVA with a post hoc Holm-Bonferroni was used to compare means, where ***p<0.0001.

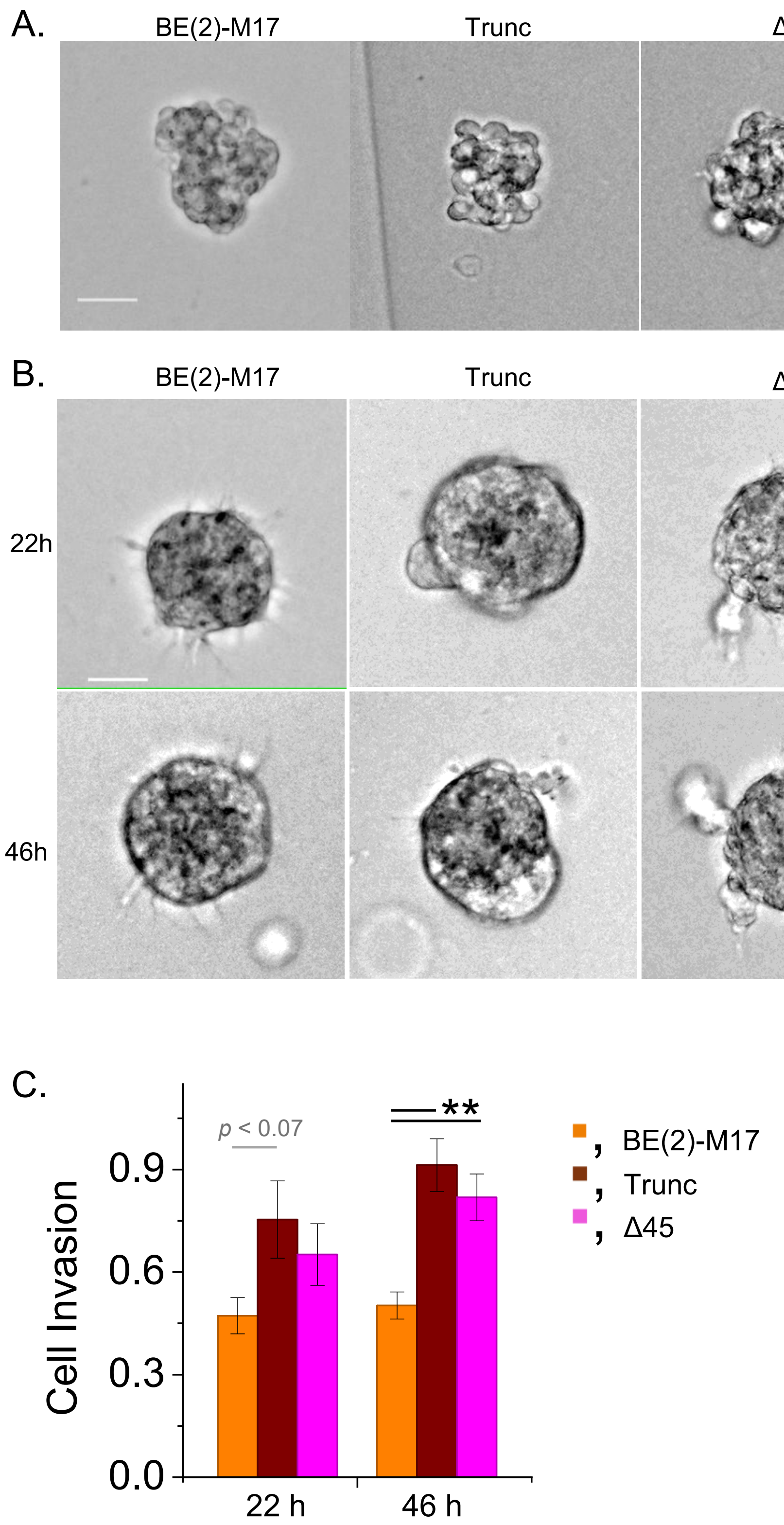

Figure 7: Loss of *MGAT1* promotes cel
A) Typical images of pre-invasion spheroi
micrographs of invading cell spheroids at
three cell lines, as indicated. C) The area
spheroid from BE(2)-M17 (*n*=38, 44), Tru
(*n*=28, 54) at 22h and 46 h, respectively.
invading cell spheroids. Scale bars are 25
are represented as mean ± SEM. One-wa
hoc Holm-Bonferroni was used to compar
**p<0.01, and as indicated.

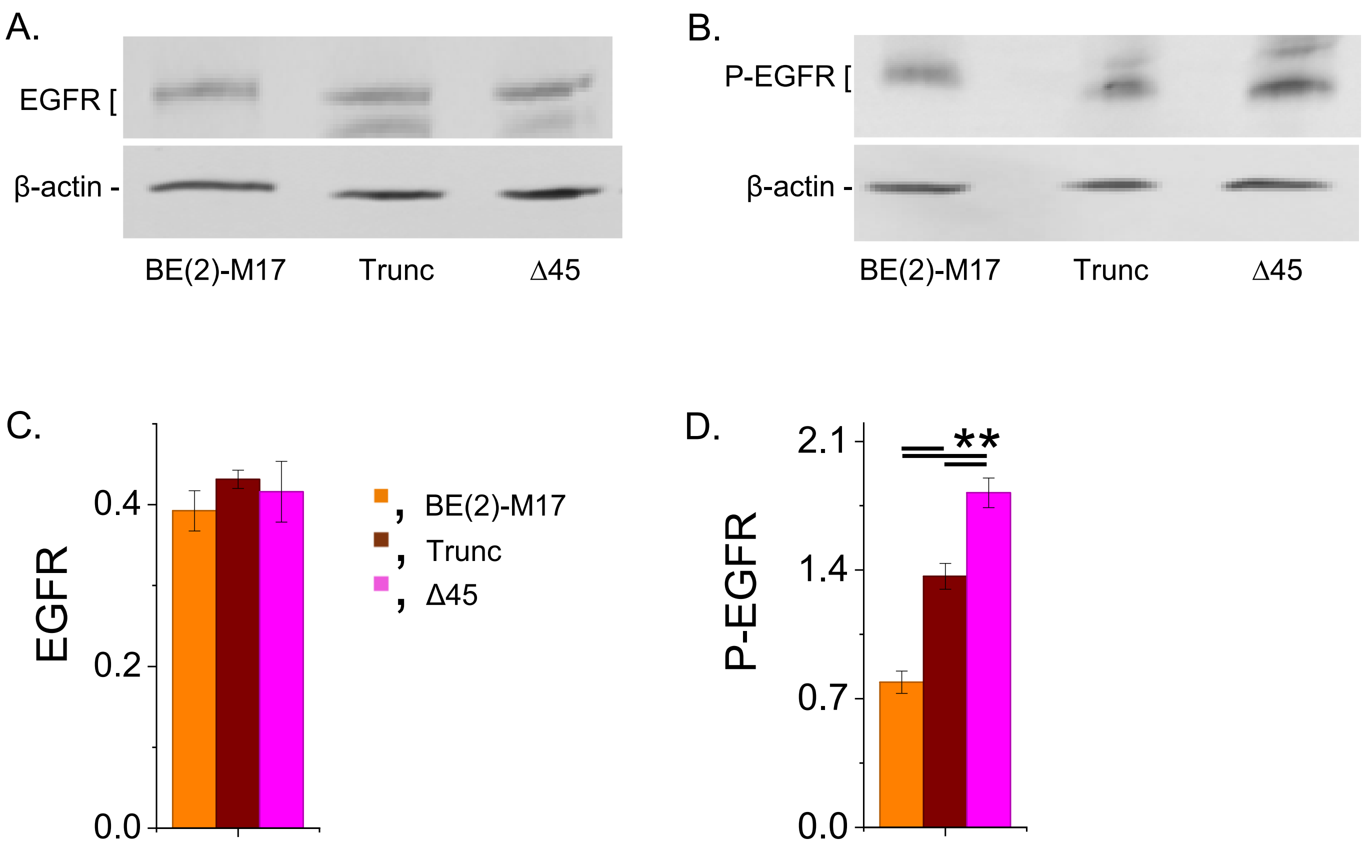

Figure 8: Reduced *MGAT1* expression alters phosphorylation of EGFR. A) Western blot of EGFR and B) phosphorylation of Y1068 in EGFR from whole cell lysates of BE(2)-M17, Trunc and Δ45. Cells were stimulated with 20 ng/mL EGF. C) Quantification of EGFR and D) phosphorylated EGFR immunobands with respect to that of β-actin for BE(2)-M17 (*n*=3), Trunc (*n*=4), and Δ45 (*n*=4), where *n* denotes number of immunobands. Quantifications are represented as mean ± SEM. One-way ANOVA with a post hoc Holm-Bonferroni was used to compare means, where $**p<0.01$. P-EGFR immunobands were unobserved in samples without EGF which were run in parallel to samples with EGF (Fig S1).

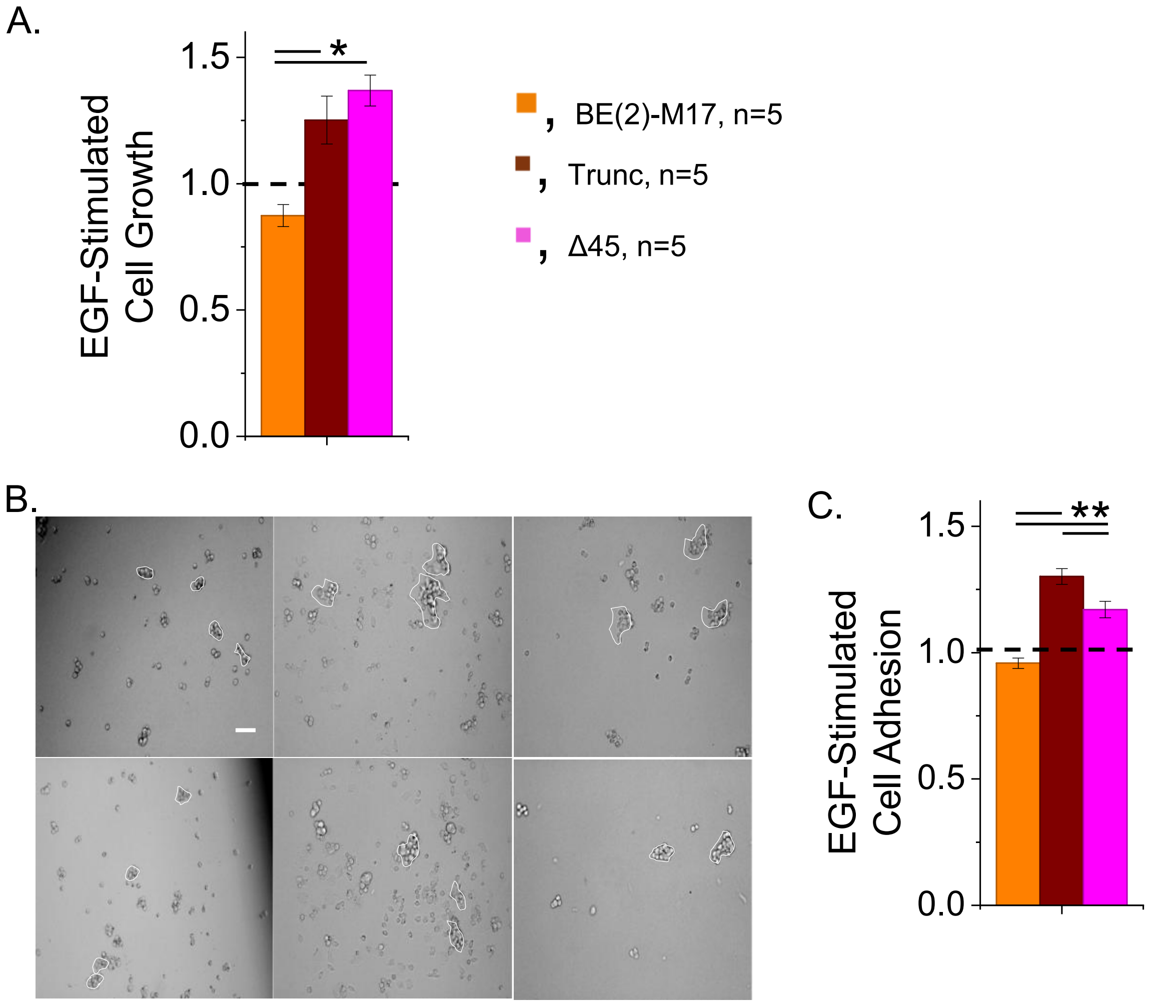

Figure 9: EGF treatment enhanced cell proliferation and cell adhesion in the *MGAT1-/-* cell lines. A) EGF-stimulated cell proliferation of BE(2)-M17 (*n*=5), Trunc (*n*=5), and Δ45 (*n*=5) where *n* is the number of replicates. B) Micrographs of BE(2)-M17, Trunc, and Δ45 cell clusters with 20 ng/mL EGF stimulation, C) and quantification of EGF-stimualed cell-cell adhesion for BE(2)-M17 (*n*=140), Trunc (*n*=175), and Δ45 (*n*=160) using 20 ng/mL, where *n* is the number of cell clusters. Scale bar is 25 μm. Data are expressed as mean ± SEM. One-way ANOVA with a post hoc Holm-Bonferroni was used to compare means from three samples and student t-test was used for two samples, where *p<0.05, **p<0.01.

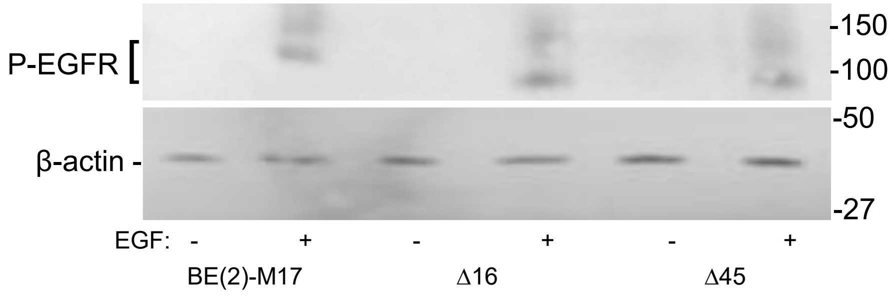

Fig S1. Western blot of the phosphorylation of Y1068 in EGFR from whole cell lysates of BE(2)-M17, Trunc and Δ45 with (+) and without (-) 20 ng/mL EGF. P-EGFR was undetected in absence of EGF.
