# Supplementary material for: Mutations in the N-glycan branching enzyme MGAT1 promote invasiveness and EGF-stimulated proliferation and adhesion in neuroblastoma cells
Source: PLoS One. 2026 Sep 10;21(9):e0358077. doi: 10.1371/journal.pone.0358077 (PMC13561367; doi:10.1371/journal.pone.0358077)

Vimentin

M17 445 447 M17 445 447 M17 445 447

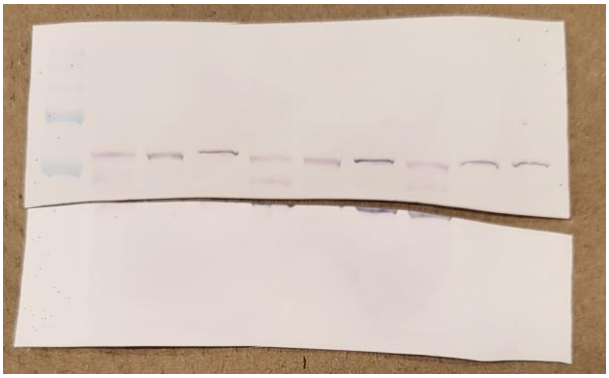

260216-RAS

447 M17 445

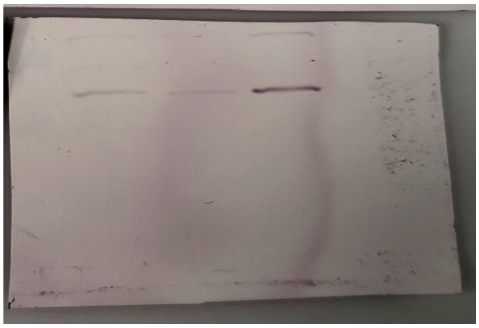

M17 445 447 M17 445 447 M17 M17 M17

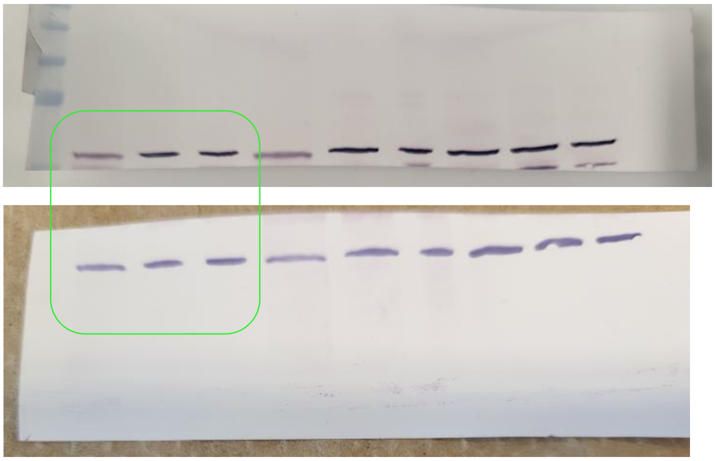

260513-MKH

M17 445 447

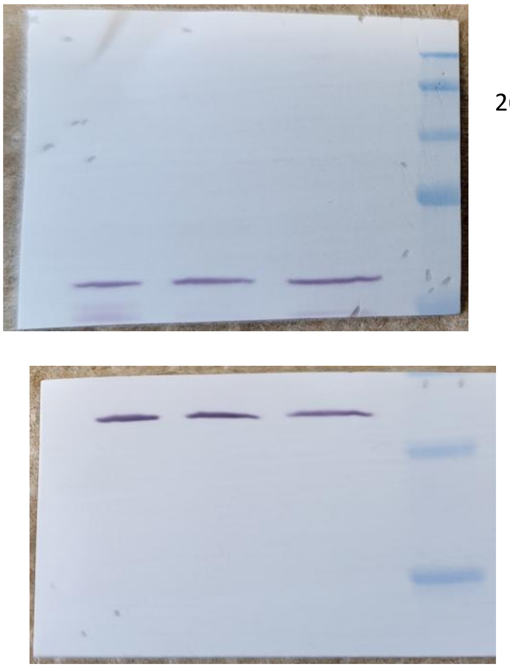

260604-MKH

EGFR

Trunc M17 Trunc M17

260603

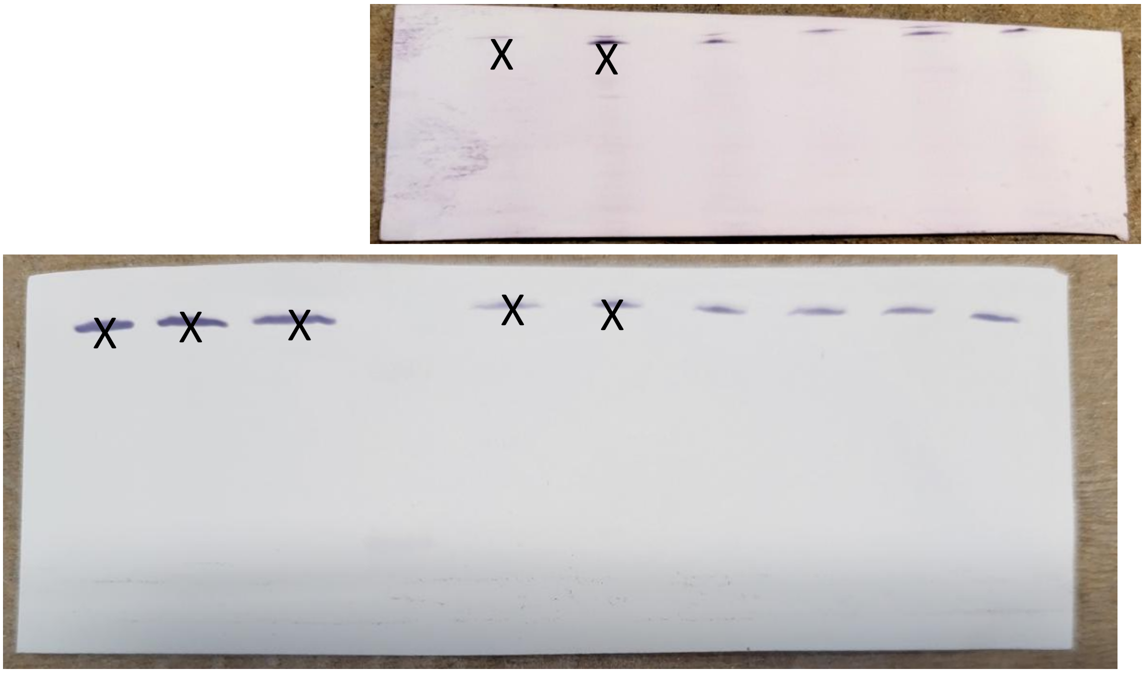

260617

Trunc M17 Trunc Δ45 M17 Trunc Δ45 M17

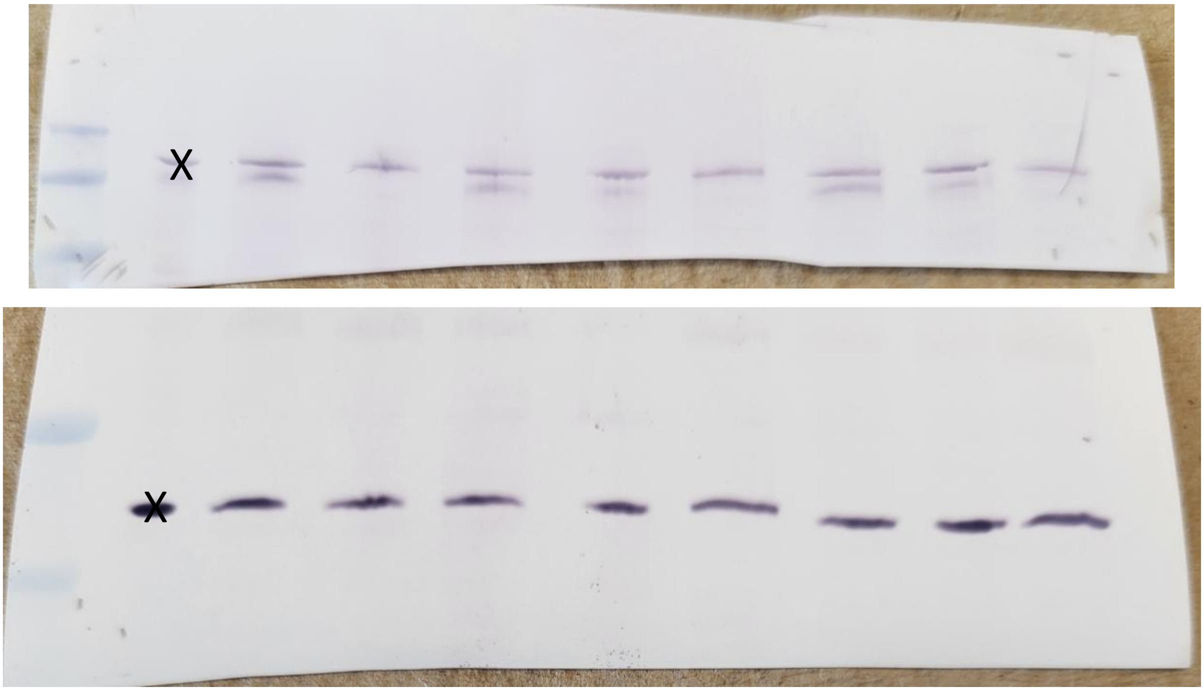

pEGFR-Y1068

M17 Δ16 Δ45 M17+ Δ16+ Δ45+ M17+ Δ16+ Δ45+

Y1068

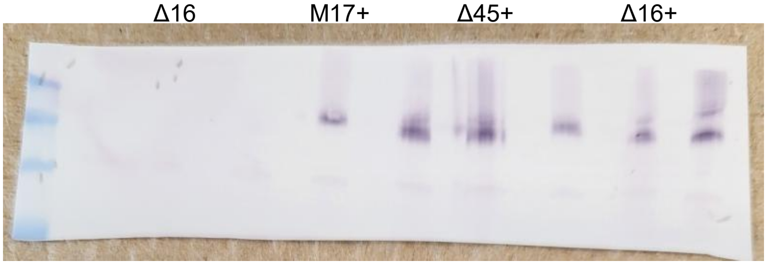

260429
20ng/mL

Actin

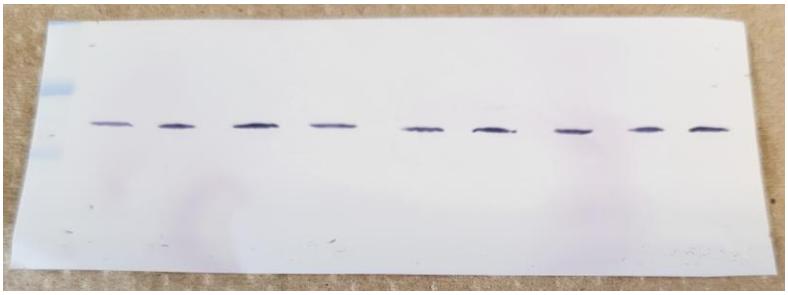

M17 M17+ Δ16 Δ16+ Δ45 Δ45+ M17+ Δ16+ Δ45+

Y1068

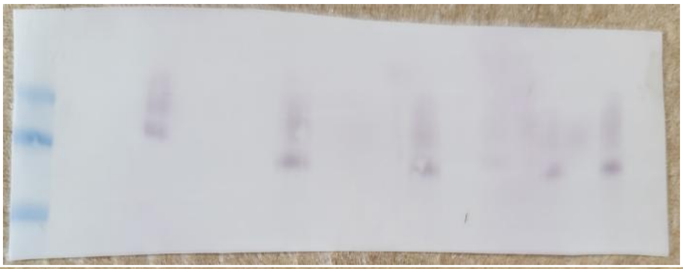

260505
20ng/mL

Actin

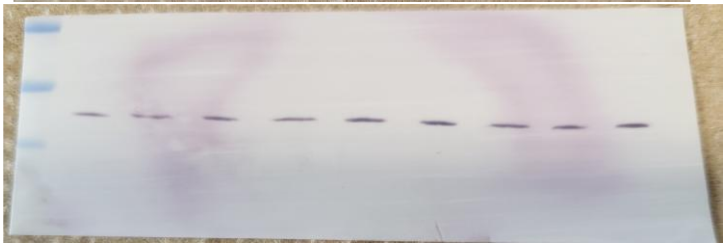

GNL

M17 M17
Δ45 Trunc Δ45

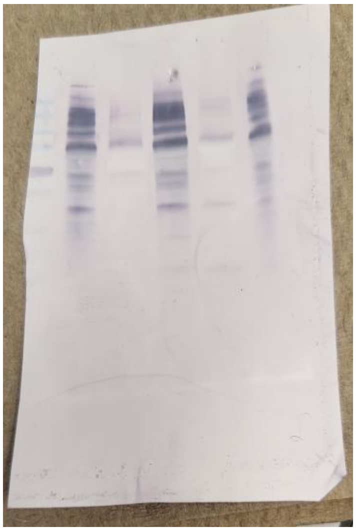

260324

coomassie

Trunc M17
Δ45

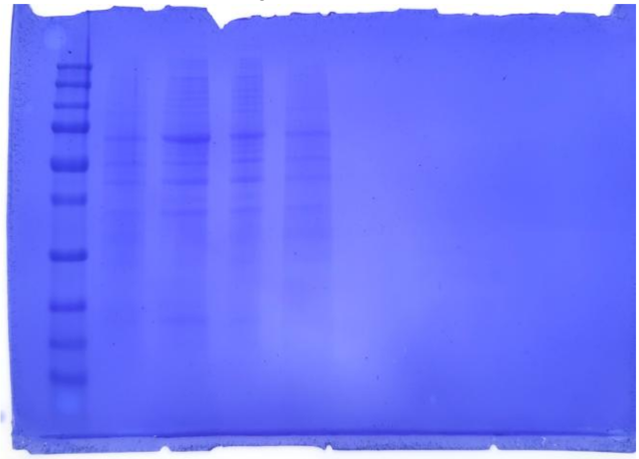

260313

GNL

Δ45 Trunc M17

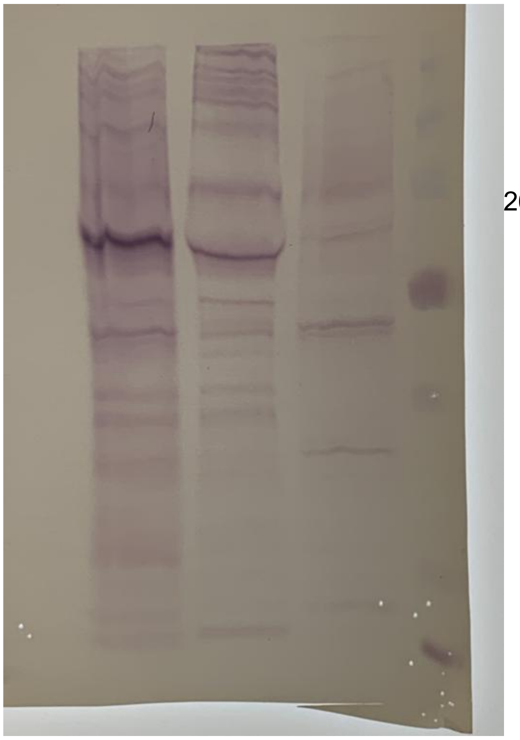

260122

coomassie

Δ45 Trunc M17

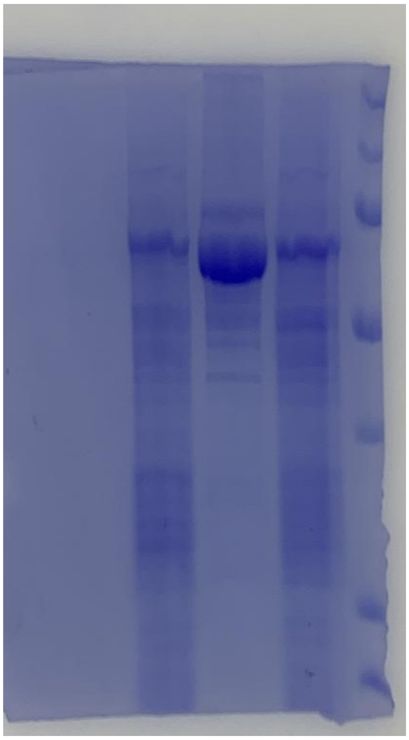

260122

GNL

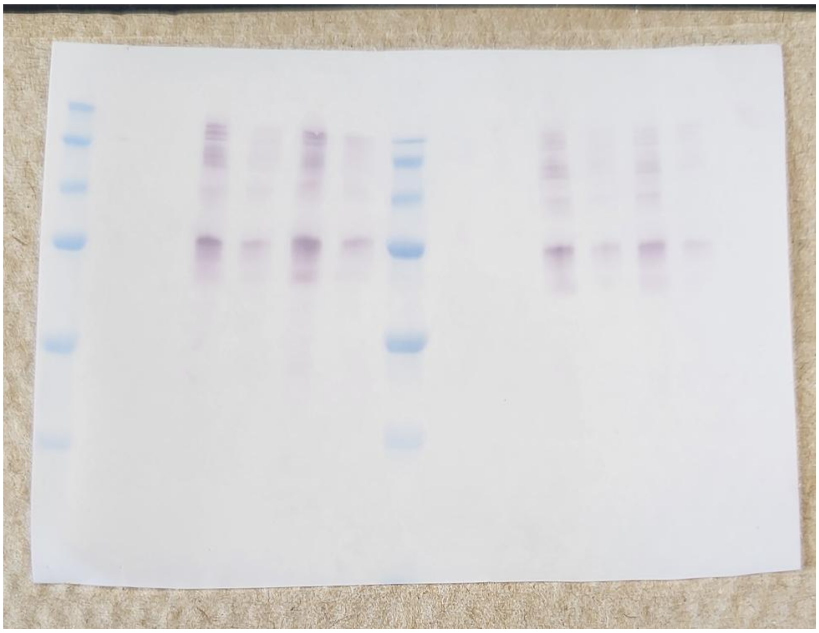
M17
+GnTI
Trunc
+GnTI
Δ45
+GnTI
M17
+GnTI
Trunc
+GnTI
Δ45
+GnTI

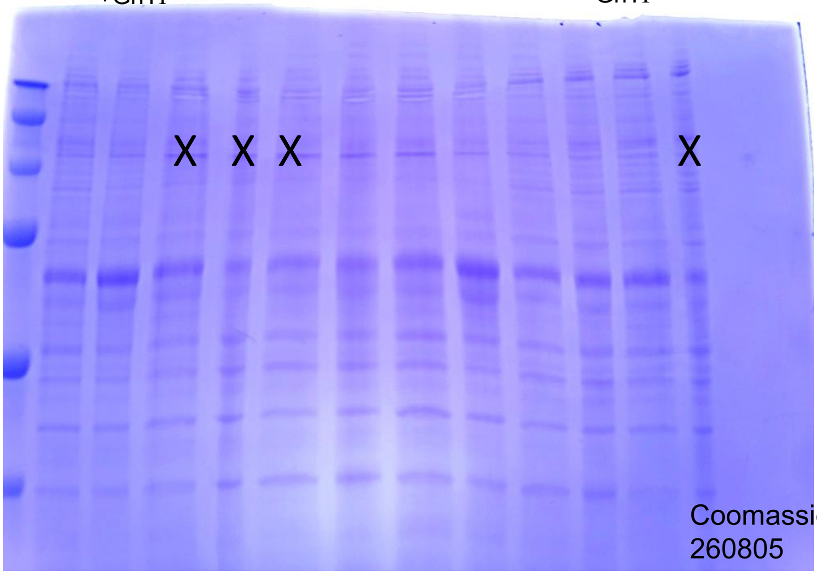
M17
+GnTI
M17
+GnTI
Trunc
+GnTI
Δ45
+GnTI
X
X
X
X
Coomassie
260805

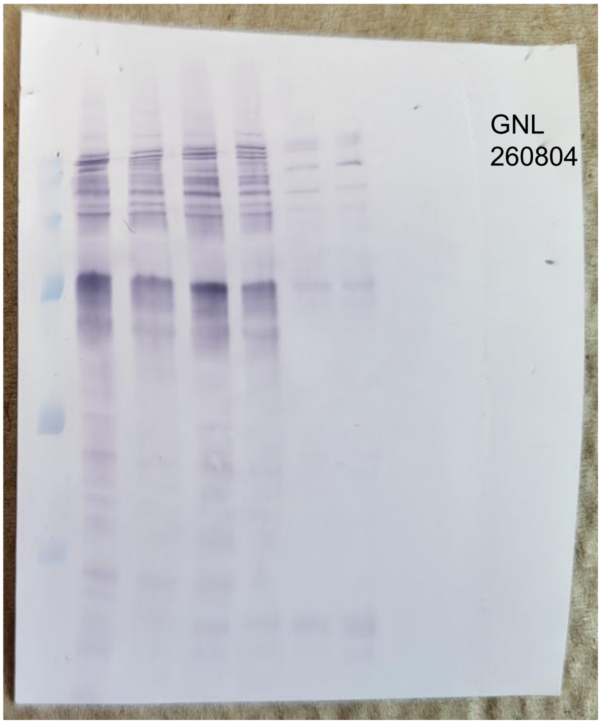
Δ45
+GnTI
Trunc
+GnTI
M17
+GnTI
GNL
260804

L-PHA

M17 Trunc M17 Δ45 M17

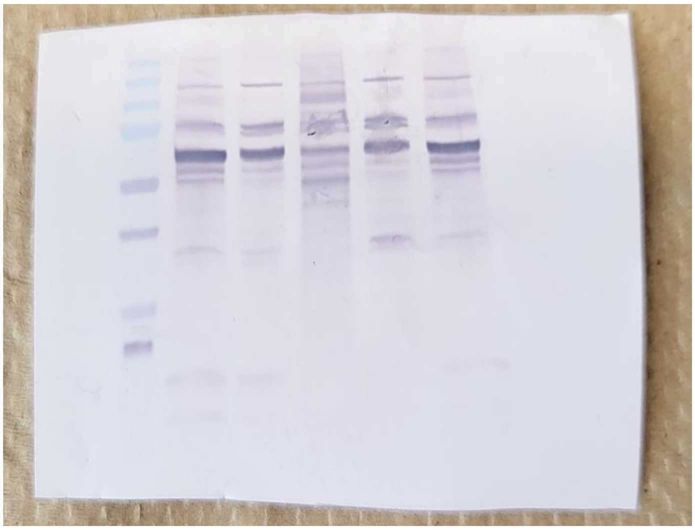

coomassie

260401

Trunc Δ45 M17

260313

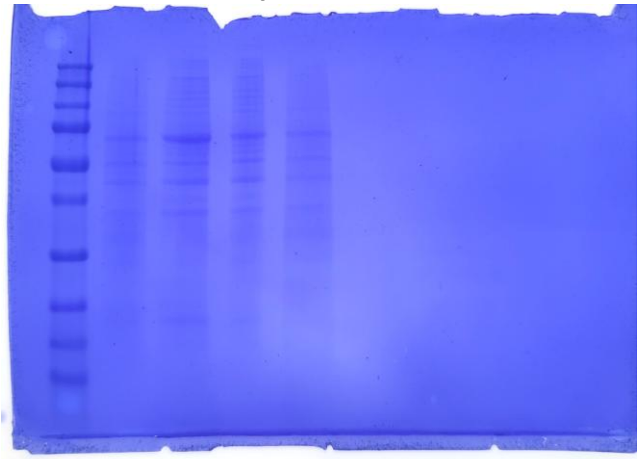

L-PHA

M17 Trunc Δ45

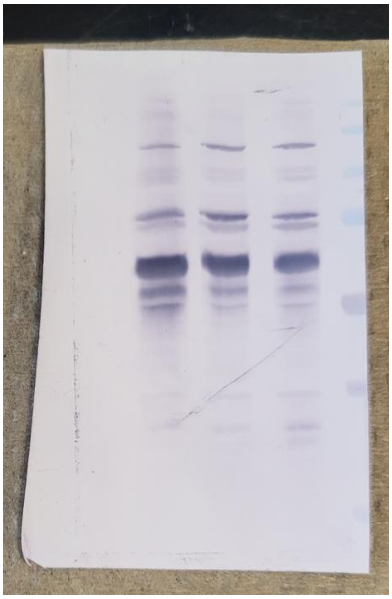

260518

coomassie

M17 Trunc Δ45

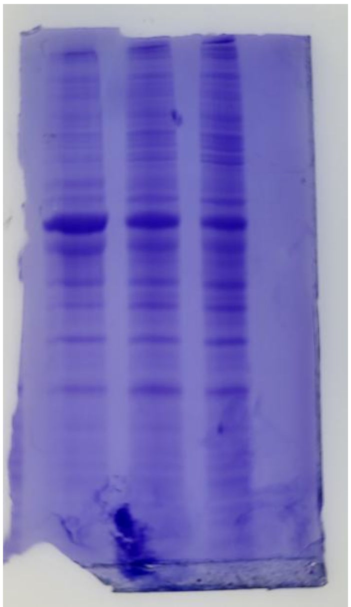

260518

L-PHA

M17 Trunc Δ45

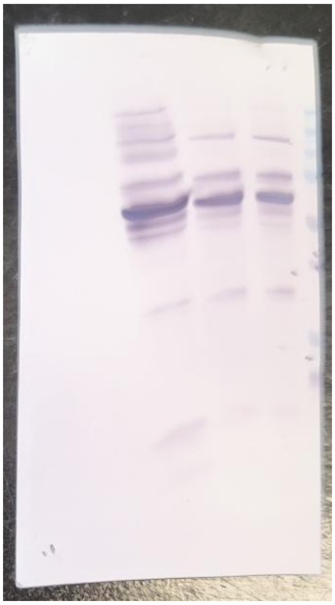

260527

E-PHA

M17 Δ45 M17 Trunc

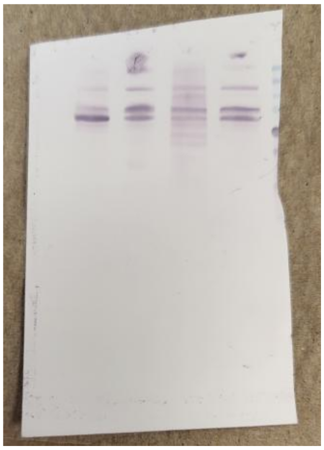

260324

coomassie

Trunc Δ45 M17

260313

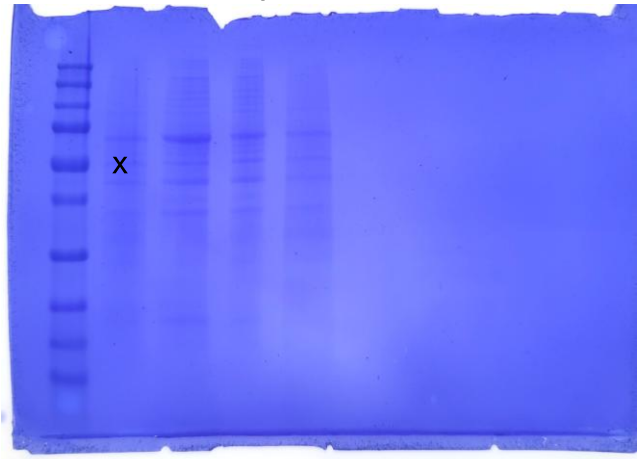

E-PHA

M17 Trunc Δ45

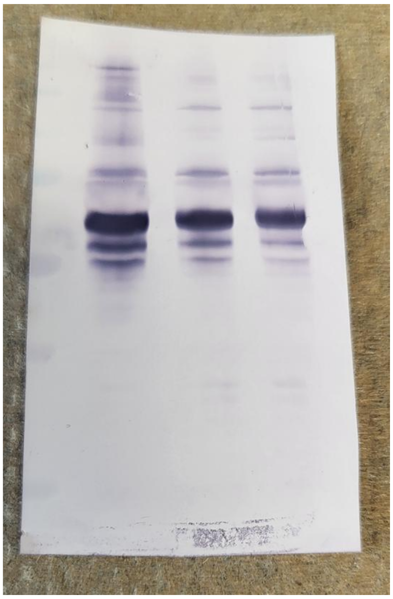

260518

coomassie

M17 Trunc Δ45

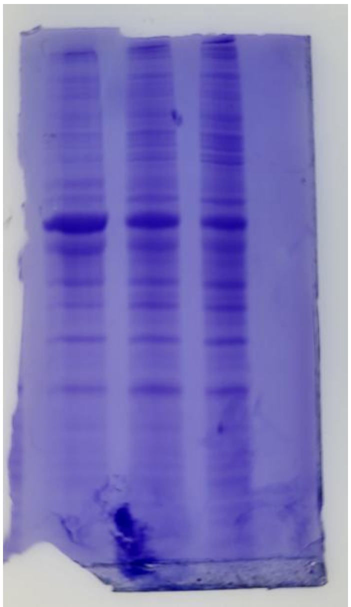

260518

M17 Trunc Δ45

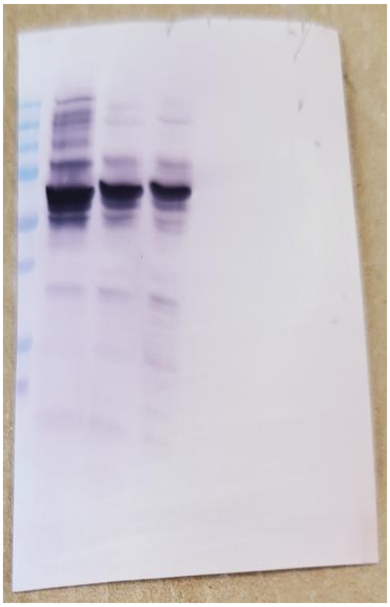

Supplement: Supplementary file 1 [file pone.0358077.s001.zip › original Lectins&WB_260808.pdf]
